# The screening, identification, design and clinical application of tumor-specific neoantigens for TCR-T cells

**DOI:** 10.1186/s12943-023-01844-5

**Published:** 2023-08-30

**Authors:** Jiangping Li, Zhiwen Xiao, Donghui Wang, Lei Jia, Shihong Nie, Xingda Zeng, Wei Hu

**Affiliations:** 1 Division of Thoracic Tumor Multimodality Treatment, Cancer Center, West China Hospital, Sichuan University, Chengdu, 610041 People’s Republic of China; 2 Department of Otolaryngology Head and Neck Surgery, The Sixth Affiliated Hospital, Sun Yat-Sen University, Guangzhou, 510655 People’s Republic of China; 3 Department of Radiation Oncology, The Third Affiliated Hospital Sun Yat-Sen University, Guangzhou, 510630 People’s Republic of China; 4 International Health Medicine Innovation Center, Shenzhen University, Shenzhen, 518060 People’s Republic of China; 5 Department of Radiation Oncology, West China Hospital, Sichuan University, Cancer Center, Chengdu, 610041 People’s Republic of China; 6 Department of Parasitology of Zhongshan School of Medicine, Sun Yat-Sen University, Guangzhou, 510080 China; 7 Division of Vascular Surgery, Sichuan Academy of Medical Sciences & Sichuan Provincial People’s Hospital, School of Medicine, University of Electronic Science and Technology of China, Chengdu, 610072 People’s Republic of China

**Keywords:** TSA, Neoantigen, TCR-T, ACT, Immunotherapy

## Abstract

Recent advances in neoantigen research have accelerated the development of tumor immunotherapies, including adoptive cell therapies (ACTs), cancer vaccines and antibody-based therapies, particularly for solid tumors. With the development of next-generation sequencing and bioinformatics technology, the rapid identification and prediction of tumor-specific antigens (TSAs) has become possible. Compared with tumor-associated antigens (TAAs), highly immunogenic TSAs provide new targets for personalized tumor immunotherapy and can be used as prospective indicators for predicting tumor patient survival, prognosis, and immune checkpoint blockade response. Here, the identification and characterization of neoantigens and the clinical application of neoantigen-based TCR-T immunotherapy strategies are summarized, and the current status, inherent challenges, and clinical translational potential of these strategies are discussed.

## Key points

1) Established the computational process and platform of neoantigen prediction, and comprehensive prediction and screening of tumor specific neoantigens.

2) Rapid identification of neoantigen-specific TCRs for personalized engineered TCR-T cell therapy.

3) Classification and characteristics of tumor specific neoantigen-based therapy, and combined antitumor strategies.

4) The current status, inherent challenges, and clinical translational potential of TCR-T therapy.

## Background

**Fig. 1:**
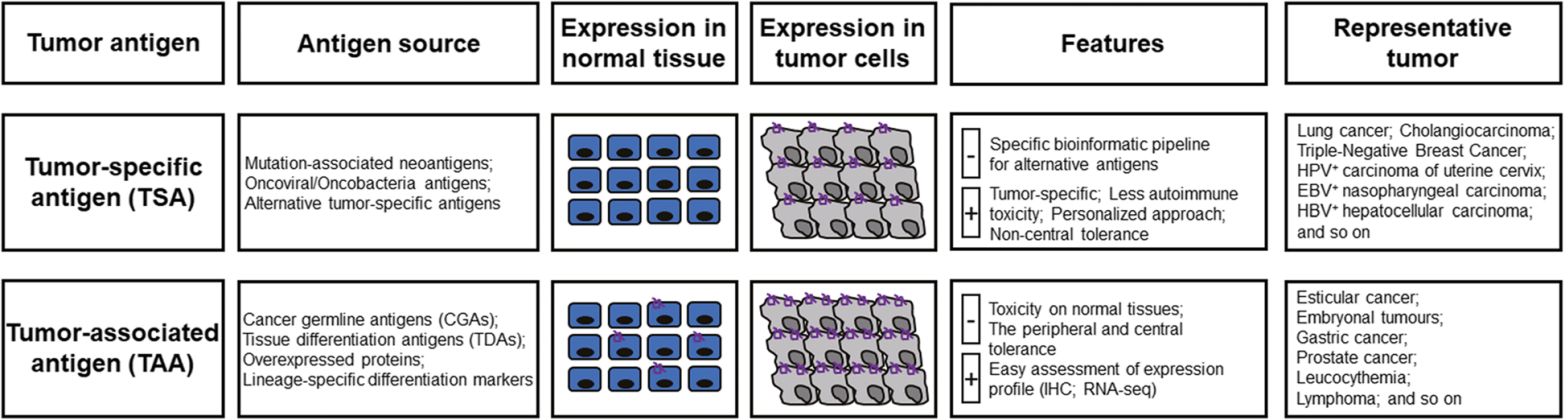
Characteristics of tumor-specific antigens (TSAs) and tumor-associated antigens (TAAs). Sources of TSA and TAA. In contrast to normal tissues, TSA is only expressed in tumors. TAA is underexpressed in normal tissues and overexpressed in tumors. The advantages and disadvantages of each antigen type of TSA and TAA and its representation of related tumors are shown

Major histocompatibility (MHC) molecules of tumor cells can express TSAs or TAAs. TSAs are abnormally expressed in malignant tumor cells or only produced at specific stages of differentiation, and its production is extremely limited in normal tissues. TAAs mainly refer to the antigenic molecules present on normal cells or tumor cells; however, they are not unique to tumor cells. Normal cells can be synthesized in small amounts and are usually highly expressed when tumor cells proliferate (Fig. 1). These tumor-specific polypeptide-MHC (pMHC) complexes are recognized by T cells and trigger an antitumor immune response in the patient. Compared with other types of tumor antigens, such as cancer-testicular antigen (CTA) and TAAs, highly immunogenic and tumor-specific TSAs provide new targets for personalized tumor immunotherapy and can be used as prospective indicators for predicting tumor patient survival, prognosis, and immune checkpoint blockade response. Recent advances in TSA research have accelerated the development and clinical trials of tumor immunotherapies, including adoptive cell therapies, cancer vaccines, and antibody-based therapies. Rapid identification, prediction, and clinical application of TSAs are made possible by advances in next-generation sequencing and bioinformatics.

Various clinical studies have examined the effectiveness of immunotherapies targeting TAAs, such as vaccines against ERBB2, MUC1, and hTERT [1–3]. The prevalence of TAAs among cancer patients makes them a target for quick immunization. Since TAAs are nonmutated autoantigens, central T-cell tolerance may be one of the reasons for the poor T-cell response observed in clinical trials [4]. However, immune escape mechanisms can be reversed by cancer immunotherapy, including the use of tumor vaccines to improve antigen presentation, the increase in antitumor T cells by adoptive metastasis of tumor-infiltrating lymphocytes (TILs) and T-cell receptor (TCR)-transduced T cells, the restoration of CD8^+^ T-cell effector capacity by immune checkpoint blockades (ICBs), and T cells induced by bispecific antibodies (bsAbs) and chimeric antigen receptors (CARs) to increase the immune recognition of tumors [5–7]. Nevertheless, widespread use of tumor immunotherapy is hampered by a shortage of antigens that target various cancers [5]. In non-virus-associated malignancies, TSAs can be derived from unique proteins or peptides that are produced by abnormal RNA splicing and disrupted posttranslational protein modification. For virus-associated cancers, such as HPV-positive cervical cancer and EBV-associated nasopharyngeal carcinoma, TSAs can also be generated through the virus-encoded open reading framework (ORF) [8, 9]. This ability to avoid T-cell tolerance increases the TSA-specific T-cell pool and thus has the potential to enhance the tumor-specific immune response. In addition, immunotherapy enhanced the tolerance of TSA-specific T-cell responses, and the immune memory after treatment offers promise for long-term protection against disease recurrence.

### 一、The source of tumor-specific antigens (TSAs)

**Fig. 2:**
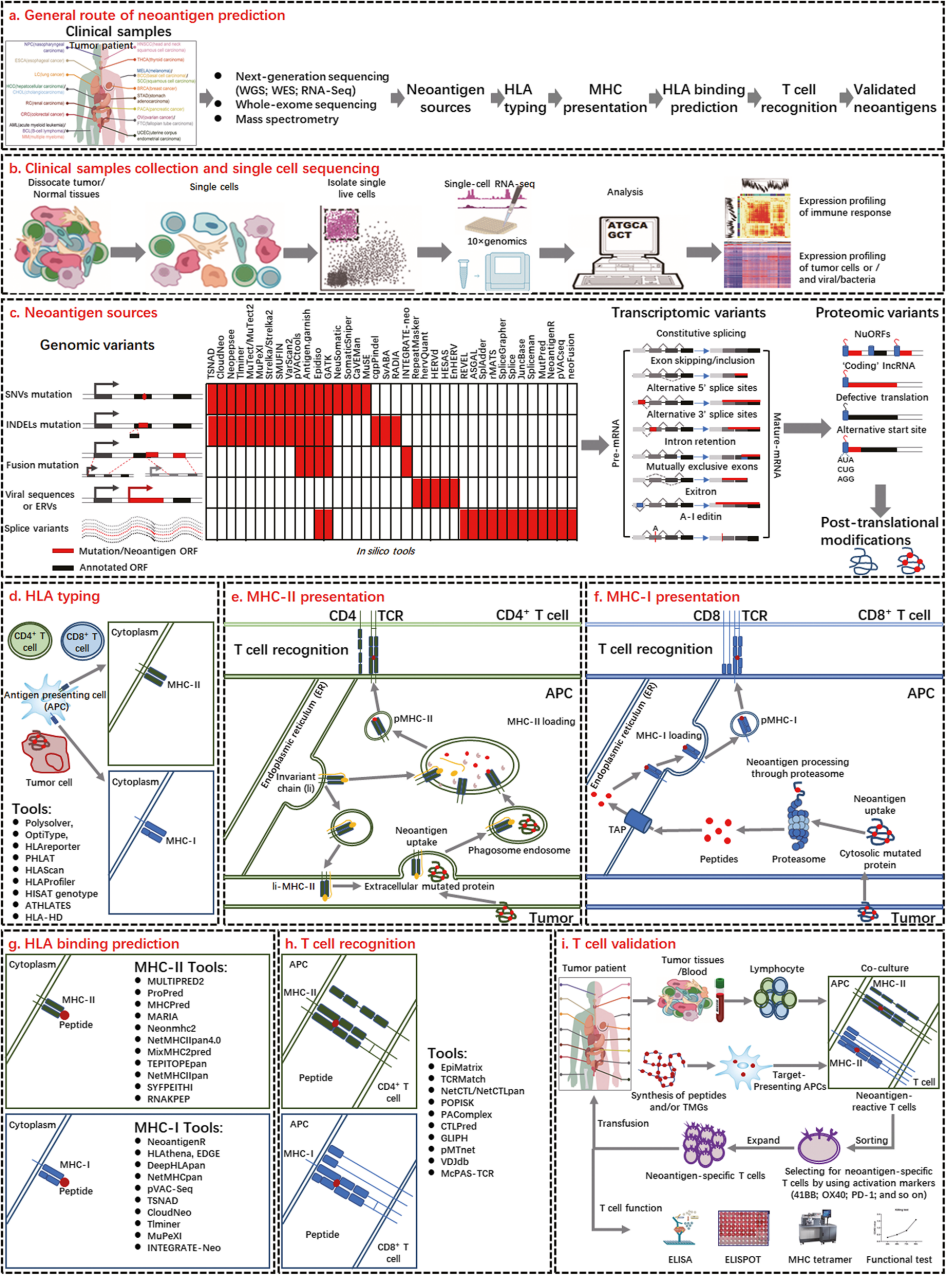
Computational workflow of neoantigen prediction. **a** The general route of neoantigen prediction. **b** The workflow of clinical sample collection and single-cell sequencing. **c** The neoantigen sources can develop at the genomic level through SNV mutation, INDEL mutation, fusion mutation, integrated viral ORF and splice variants (the display of prediction software for mutation calling), at the transcriptomic level through alternative splicing, polyadenylation (pA), RNA editing and allegedly noncoding regions, and at the proteomic level through dysregulated translation and PTMs. **d** HLA typing prediction and display by prediction software tools. **e** and **f** Mutant polypeptides are produced by proteasome-mediated decomposition of endogenous proteins, which are subsequently transported to the ER by antigen-processing associated transporters (TAP). They may be loaded into MHC-I and MHC-II for binding to specific peptides produced by mutated proteins that breakdown in the endosomal pathway. These peptide-MHC-II/MHC-I (pMHC) complexes are then transported to the cell surface, where they are recognized by T cells. **g** pMHC complex binding prediction and the display of prediction software tools. **h** The prediction of T-cell recognition of pMHC complexes and the display of prediction software tools. **i** T-cell validation of neoantigens. Coculture of patient TILs or PBMCs with autologous antigen-presenting cells (APCs) expressing candidate neoantigens (TMG or peptides) allows for the identification of neoantigen-reactive T cells based on functional data such as IFN-γ release or 4-1BB expression. On the one hand, it could be injected into patients for cell therapy. On the other hand, the related functions of neoantigen reactive T cells have been verified by different experiments

TSAs are produced due to changes in the genome, transcriptome, and proteome. They are foreign proteins that are not present in normal tissues but can be produced from tumor cells through various mechanisms, such as genomic mutations, abnormal transcriptional mutations, posttranslational modification (PTM) mutations, and viral-encoded open reading framework (ORF) mutations [10] (Fig. 2a, b, c; Table 1).

**Table 1:** The advantages and disadvantages of various neoantigens and associated cancers

Neoantigen classification	Sources	Advantages	Disadvantages	Cancers
**Genomic variants**	Single-nucleotide variants (SNVs) mutation	Relatively high burden; Simple prediction	Rarely shared between patients; similar to self-antigen	Bladder cancer; Lung cancer (squamous and aseno); Melanoma; Glioblastoma
	Insertions and deletions (INDELs) mutation	More immunogenic; More potential targets per mutation; More dissimilar from self-antigen	Relatively low burden	Renal cell carcinomas (clear cell, chromophobe and papillary); MSI-H tumors
	Genes fusion mutation	More immunogenic; More potential targets per mutation; More dissimilar from self-antigen; Shared targets between tumors	Relatively low burden	Chronic myeloid leukemia; Acute lymphocytic leukemia; Acute myelocytic leukemia; Sarcomas
	Structural variants (SVs) mutation	High immunogenicity	Less well studied	Malignant pleural mesothelioma
**Transcriptomic variants**	Transcript alternative splicing	High immunogenicity; A large number of predicted targets	Less well studied	Squamous cell carcinoma of pancreas; Adenocarcinoma of lung
	Polyadenylation (PA) and RNA editing	Easy predication	Less well studied	Chronic lymphocytic leukemia
	Allegedly non-coding regions	More potential targets; Relatively high burden	Less well studied; Fewer tools available	Acute lymphoblastic leukemias; Lung cancers
	RNA splicing	More dissimilar from self-antigen; A large number of predicted targets	Fewer tools available; Current tools do not account for nonsense mediated decay (NMD); Not well validated in pre-clinical models;	Chronic lymphocytic leukemia; Acute myelocytic leukemia; Chronic myelocytic leukemia; Myelodysplastic syndrome
**Proteomic variants**	Post-translational modifications (PTMs)	Shared between patients	Less well studied	Non-small cell lung ancer; Leukemia; Renal cancer
	T cell epitopes associated with impaired peptide processing (PEIPP)	TEIPP-specific T cells can escape thymic selection	Less well studied; TAP-deficient tumors or Limited in HLA-I low	Lung cancer
	Transporters associated with antigen processing (TAP)	High specificity	Less well studied; Fewer tools available	Acute myelocytic leukemia
**Viral-derived neoantigens**	Viral open reading frames (ORFs) or viral sequences or ERVs	High immunogenicity; Shared between patients; More dissimilar from self-antigen; Without apparent toxicity to normal tissues	Limited in specific tumors	Head and neck cancer; Merkel cell carcinoma; Nasopharyngeal carcinoma; Cervical cancer; Hepatocellular carcinoma; Anal cancers
**Tumor-testicular antigen**	Testis or placenta	High immunogenicity; Strong immunogenicity; Not expressed in normal tissues except testicles and placenta	Less well studied; Limited in specific tumors	Testicular cancer; Germ cell tumor
**Tissue differentiation antigens and others**	Melanoma or melanocytes or polysaccharides or lipid antigens	Most are presented by HLA-A2	Less well studied; Cause damage to normal tissue; Inability to effectively present autoantigens	Melanoma; Some gastrointestinal tumors

### (一)、Neoantigens derived from genomic variation

Up to hundreds of nonsynonymous somatic mutations have been recorded in cancer patients, resulting in an average of 150 potential neoantigens per person. Somatic genome changes, including single nucleotide variants (SNVs), base insertions and deletions (INDELs), gene fusion, and structural variants, are major factors that promote TSA production [11].

#### Single nucleotide variations (SNVs)

SNVs are the most common type of mutation at the genomic level of tumor cells, and they can produce mutant peptides distinct from wild-type peptides, which are presented by the MHC as specific antigens [12]. The neoantigen profiles encoded by SNVs vary greatly between different cancer types and different stages of the same cancer type. Using the TCGA database, 933954 neoantigens were identified in 20 solid tumors. These mutations resulted from somatic mutations with different median frequencies of 893960 neoantigens in different cancers. At least 5% of patients with the same or different cancers share 24 of these neoantigens, including those caused by driver gene mutations such as PIK3CA, RAS and BRAF [13]. SNVs can also appear in mitochondrial DNA (mtDNA). mtDNA is present in most cancer cells and is involved in tumor metabolism and metastasis. Both mouse and human immune systems can recognize and respond to mtDNA-SNP-derived peptides, suggesting that a single SNP in mtDNA is sufficient to produce immunogenic antigens [14]. Thus, nonsynonymous SNPs in mtDNA may produce a large number of mutant peptides, providing an additional source of neoantigens.

#### Insertions and deletions (INDELs)

INDEL mutations are mainly caused by the insertion or deletion of base pairs in the genome, which frequently lead to nonsynonymous novel ORFs, also known as frameshift mutations. Frameshift INDELs can generate more types of neoantigens with increased MHC-I binding affinity, suggesting a higher immunogenic mutation type compared to SNVs [10]. For example, in renal cell carcinoma with moderate mutation loads, approximately 16% of predicted new peptides came from frameshift insertion, while 21% of new epitopes recognized by T cells came from frameshift insertions [15], suggesting greater immunogenic potential for frameshift-derived epitopes. Similar to SNV-encoded neoantigens, INDEL-encoded neoantigens are more common in cancers with high microsatellite instability (MSI-H), which is determined by the absence of DNA mismatch repair (MMR) mechanisms. As the evolution of MMR-deficient cancers is mainly triggered by mutations that inactivate tumor suppressor genes (TSGs) containing coding microsatellites, frameshift peptide neoantigens are more frequently shared among MMR-deficient cancers (e.g., endometrial, colorectal, and gastric) than missense mutation-derived neoantigens [16]. Frameshift mutation-encoded neoantigens are more common than missense mutation-derived neoantigens in MMR-deficient cancers, such as breast, colorectal, gastric, and endometrial cancers, and have a strong association with immune responses and a larger proportion of TILs; thus, these neoantigens are excellent candidates for immunotherapy targeting MSI cancers. MSI colorectal cancers with frameshift mutations have a larger proportion of TILs than other colorectal cancers. Similarly, shared immunogenic frameshift peptide neoantigens can be produced as a result of recurrent frameshift mutations, offering excellent candidates for immunotherapy against MSI cancers [17–20]. The results showed that the combination of four frameshift-derived polypeptides could significantly improve the acquired immune response of mice in a VCMsh2-driven intestinal tumor model, reduce the intestinal tumor burden, and prolong overall survival [21]. According to clinical phase I/IIa trials, frameshift mutation-encoded neoantigen vaccines have good systemic tolerance and regularly trigger immune responses, providing a promising new strategy for the treatment and even prevention of MMR-deficient malignancies [22]. These findings suggest that off-the-shelf frameshift mutation neoantigen-driven vaccines are feasible options for the treatment and prevention of MSI cancers. In addition, the frameshift INDEL neoantigen burden is also a novel biomarker for ICB response. INDEL-frameshift mutations are thought to produce more immunogenic neoantigens, thereby improving the response to ICB therapy; thus, these mutations can be novel biomarkers for the ICB response. INDEL frameshift mutations are supposed to produce more immunogenic neoantigens, hence improving response to ICBs. When frameshift mutations are present, the progression-free survival of patients receiving ICBs is significantly prolonged [23]. For example, considerable variation in overall response rates and disease control rates has been observed in patients with frameshift-mutated non-small cell lung cancer (NSCLC). Progression-free survival (PFS) was significantly prolonged in patients treated with ICBs when frameshift mutations were present [24]. Therefore, ICBs enhance the immune response to frameshift-driven neoantigens. Pembrolizumab blocks PD-L1 in vivo, restoring frameshift neoantigen-specific T-cell immunity [25].

#### Gene fusion

Chromosomal translocations or deletions induce DNA rearrangements that can lead to gene fusion, which may produce fusion proteins, another important type of mutation in tumors that may provide many neoantigens [26]. For example, the BCR-ABL fusion protein is produced by translocations between chromosomes 9 and 22 in patients with chronic myelogenous leukemia (CML) [27], and the SYT-SSX1 fusion protein is produced by translocation of chromosomes X and 18 in patients with synovial sarcoma [28]. A further expanded study of 30 different tumor types in the TCGA database found that 24% of cancers expressing fusion proteins contained new epitopes produced by fusion, which were predicted to bind to patient-specific MHC, and peptides from different fusion regions were recognized by the patient's own T cells. At the same time, only 5.8% of fusion-driven neoantigens were consistent between patients, and the immunogenicity of these neoantigens was usually low [29, 30]. A comprehensive analysis of three TCGA databases found that fusion mutations produced more ORFs and approximately 6 and 11 times more neoantigens than SNV and INDEL mutations, had better immunogenicity, and induced a stronger immune response. Similar to the candidate neoantigen load produced by SNV and INDEL mutations, fusion neoantigen load is closely related to fusion mutation load, especially in microsatellite stable tumors with high fusion mutation load [31, 32]. In addition, immune depletion microenvironments or malignancies with HLA loss are more likely to present fusion neoantigens. According to FACETS analysis of the TCGA exome data, HLA heterozygosity loss (LOH) occurred in 18.4% of the cases, which increased the likelihood of the presence of fusion neoantigens [33]. For example, in melanoma treated with anti-PD-1, the clearance of tumor cells carrying fusion-derived neoantigens showed negative immune surveillance selection pressure against these neoantigens [34]. Neoantigens produced by gene fusion are still able to activate cytotoxic T cells even in some tumors with low TMB and limited immune penetration [29]. These results suggest that gene fusion is important as a source of tumor-specific neoantigens.

Neoantigens derived from gene fusion can induce tumor-specific immune responses. A comprehensive study of 33 tumor types found a variety of common fusion neoantigens, including TMPRSS2-ERG, MYB-NFIB, EML4-ALK, DEK-AFF2, CBFB-MYH11, and SYT-SSX [35–38]. TMPRSS2-ERG is the most common recurrent fusion gene, occurring in 38.2% of prostate cancer patients. Several HLA-restricted epitopes with high affinity were identified from relapsed TMPRSS2-ERG type VI fusion, which bind to HLA-A*02:01 in vitro and are recognized by CD8^+^ T cells [39, 40]. Fusion of the proto-oncogene MYB with the transcription factor NFIB is a biomarker of adenoid cystic carcinoma and occurs in 60% of cases [41]. One NFIB-MYB-derived peptide (MMYSPICLTQT) and three MYB-NFIB-derived peptides (QFIDSSWYL, SLASPLQPT, and SLASPLQSWYL) bind to HLA-A*02:01 to activate the immune system [29, 42]. The EML4-ALK fusion gene is mainly present in 5% of young NSCLC patients who either rarely smoked or never smoked. The use of EML4-AlK-derived peptides can stimulate specific CTL responses and potentially treat EML4-AlK-positive NSCLC patients [43, 44]. A study of head and neck squamous cell carcinoma (HNSCC) found that the immune response to PD-1 treatment is mediated by neoantigens produced by DEK-AFF2 fusion. DEK-AFF2-derived peptides (DKESEEEVS) enhance T-cell activation according to MHC class when delivered to autologous peripheral blood mononuclear cells (PBMCs) [45]. The CBFB-MYH11 fusion antigen is distributed on acute myeloid leukemia (AML) cells and can activate T cells to induce specific killing of tumor cells [46]. SS393 (GYDQIMPKK) and SS391 (PYGYDQIMPK) are two common SYT-SSX fusion antigens in synovial sarcoma. These neoantigen peptides successfully induce synovial sarcoma-specific CTLs, which specifically kill HLA-A24-positive synovial sarcoma cells containing SYT-SSX neoantigens and target cells with peptide pulses [47, 48]. In addition, FGFR3-TACC3, CCDC6-RET, BCR-ABL, SYT-SSX1/SSX2, PAX3-FOXO1, TPM3/TPM4-ALK, EBS-FLI1 and other fusion antigens are also immunogenic neoantigens, which provide possible targets for tumor immunotherapy [49–54]. Therefore, neoantigens produced by fusion mutations greatly increase the capacity of the tumor-specific antigen library, providing more potential targets for cancer immunotherapy.

#### Structural variants (SVs)

SVs are one of the most common forms of driver gene mutations in tumors that can lead to changes in genome structure and alter gene expression or function to promote the malignant transformation of tumors [55]. SVs generally refer to genetic variants greater than 50 base pairs, such as insertions, deletions, inversions, translocations, duplications/amplifications, chromosome additions and subtractions, and chromosome rearrangements [56]. For example, in patients with malignant pleural mesothelioma (MPM), potential neoantigens produced by chromosome rearrangements have been identified using a combination of MPseq and RNAseq. Rearranging associated neoantigens can produce MPM-specific immune responses in a manner similar to that of INDEL-induced neoantigens [57, 58]. In particular, neoantigens produced by SVs were predicted to be presented on MHC proteins closely associated with the clonal proliferation of TILs, and effector T cells targeting these neoantigens were found in circulating blood [59]. Therefore, SV-derived neoantigens may also be valuable targets for antitumor immunotherapy.

### (二)、Neoantigens derived from transcriptome variations

Selective processing of mRNAs, including selective splicing events, polyadenylation (pA), RNA editing, so-called non-coding events, and post-transcriptional events offer the possibility of expanding the neoantigen space and contributing to tumor-specific antigen diversity [60].

#### Transcript alternative splicing

Abnormal selective mRNA splicing is another source of tumor-specific antigens. In normal cells, RNA splicing is the efficient and realistic conversion of immature mRNA into mature RNA. However, neoantigens derived from the selective splicing of transcripts may be caused by mutations in RNA cis-regulatory elements, trans-regulatory factors, or core spliceosomes [61]. Mutations in cis-acting elements generate potential neoantigens by altering splicing. Extensive studies of TCGA have revealed many exon‒exon connections unique to tumors, most of which can express novel neoantigens [62]. Transaction changes, in which somatic mutations in splicing factors lead to splicing mutations, induce the formation of neoantigens across the genome [63]. In hematological malignancies, common mutations in spliceosomal components, including SRSF2, SF3B1, and U2AF1/2, increase the expression of splicing variant mRNA and lead to neoantigen translation and protein expression [64–66]. In cells with normal sense-mediated RNA decay (NMD) function, a first round of translation is required to initiate NMD-mediated degradation of abnormal transcripts, which may also result in the production of small amounts of neoantigens [67]. For example, mutations in the highly conserved core NMD factor UPF1 are prevalent in pancreatic and lung adenocarcinoma, and these mutations increase the frequency of abnormal transcription and neoantigen production [68, 69]. Highly abnormal splicing events in tumors increase the amount of potential tumor-specific antigens, especially in tumors with low copy number variation and somatic cell mutation rates [30, 60]. In conclusion, these studies emphasize the idea that the alternative splicing of transcripts can promote neoantigen production.

#### Polyadenylation (pA) and RNA editing

Similar to RNA splicing, polyadenylation (pA) and RNA editing can alter the proteomes of tumor cells, thereby increasing the amount of potential immunotherapeutic targets. However, it must be emphasized that because RNA editing can also occur in normal tissues, these peptides are not necessarily tumor-specific [70, 71]. In tumors, genes upstream of the new pA site and downstream of the nearest 5' splicing site are translated when a specific polyadenylation (IPA) event occurs at the coding region. By comparing RNA-seq data from tumor and normal tissue samples of different cancers, more neoantigens produced by IPA can be identified, and these neoantigens are potential targets for cancer immunotherapy [60]. Similar to splicing and IPA, RNA editing induces nonsynonymous substitution by altering specific nucleotides in the RNA sequence, which can produce new proteins that can be presented by the MHC molecule to further induce activation of specific CD8^+^ T cells. These results suggest that these novel peptides are immunogenic [72].

#### So-called non-coding events

Recent studies have shown that many regions previously defined as noncoding regions have been shown to have coding functions [60, 73]. The screening of neoantigens from exon-region mutations alone is limited in scope, given that 99% of tumor-specific mutations occur in the noncoding regions of genes and exon regions account for only 2% of the entire human genome [74]. Thus, by studying these newly defined coding genes, many new antigenic peptides that can be presented by MHC have been identified as targets for TIL immunotherapy [75]. These MHC-related peptides (MAPs) from noncoding region genes extend the range of CD8^+^ T-cell immune monitoring from 2% (the proportion of exons in the human genome) to 75% [76]. Using mass spectrometry (MS), it has been found that many noncoding regions produce large amounts of abnormally expressed tumor-specific antigens, most of which result from epigenetic modifications in atypical translation events rather than mutations. These abnormally expressed tumor-specific antigens are more common than neoantigens produced by coding mutations and can be shared between tumor patients [77–79]. Therefore, there is an urgent need to develop fast and effective computational methods to screen these potential neoantigens and verify their feasibility for use in immunotherapy.

### (三)、Neoantigens derived from proteomic variations

Due to the deletion and replacement of nucleotides encoding a protein during translation, the reading frame is shifted forward, backward or mutated. The functional abnormalities of protein posttranslational modification (PTM), proteasome processing, and antigen-processing related transporter (TAP) ultimately lead to changes in the translated protein sequence, providing an important new source of tumor-specific neoantigens [80, 81].

The presentation of neoantigens by MHC molecules to T cells maintains specific PTMs [82]. Abnormal PTMs, including glycosylation, O-linked beta-n-acetylglucosamine (O-GlcNAc), and phosphorylation, can produce neoantigens presented by the MHC complex in tumors [83]. In addition, an unusually large proportion of mutations may promote the formation of new N-glycosylation sites, leading to the production of neoantigens [84]. Abnormal phosphorylation can produce new antigens by promoting the binding of epitopes to MHC molecules or by altering the antigenicity of the presented epitopes [85]. For example, tumor-associated phosphopeptides derived from BCAR3 and IRS2 are immunogenic to healthy donors and mice [86–88]. Covalent KRAS-G12C inhibitors, such as ARS1620, produce covalently modified peptides that can be presented on MHC to stimulate T-cell responses [89]. These tumor-specific PTMs involve covalent drug-mediated alkylation of mutated cysteine residues on cancer proteins, providing a new source of neoantigens and an easy target for immunotherapy [83].

In addition, another new set of antigenic epitopes is derived from damage that occurs during proteasome processing or the TAP complex. The proteasome processes proteins and converts them into peptides, which are particularly critical for the process of converting proteins to MHC-restricted epitopes. According to preliminary statistical analysis, the proteasome is responsible for splicing approximately one-third of MHC-I-related immune peptides and is capable of activating CD8^+^ T cells [90, 91]. Splicing epitope-specific CD8^+^ T cells from the TILs of human acute myeloid leukemia (AML) patients inhibited the growth of corresponding tumor cells in severe combined immunodeficiency (SCID) mouse models [92]. Neoantigens derived from protein splicing can provide undeveloped or unidentified neoantigens for antitumor vaccines and cancer immunotherapy. However, most tumors eventually acquire resistance and immune escape mechanisms [93]. Tumors can avoid being recognized by T cells by producing defective HLA-I antigen processing pathways or by downregulating the expression of related genes [94]. A class of neoantigens known as T-cell epitopes associated with impaired peptide processing (TEIPP) has been found in some tumors with low HLA-I/TAP deficiency [95]. They are a class of unmutated antigens from the tumor's own housekeeping proteins that activate TEIPP-specific CD8^+^ T cells and specifically kill these TAP-deficient cancer cells. TEIPP peptides are currently considered immunogenic because they are not presented by normal cells, and TEIPP-specific T cells are not negatively selected for in the thymus [96, 97]. For example, a TEIPP peptide derived from the Lass5 protein, also known as Trh4, was able to activate specific T lymphocytes and inhibit the growth of MHC-I low/TAP deficient tumors in a TCR-transgenic mouse model [98, 99]. In addition, other nonmutated tumor epitopes of TEIPP, including calcitonin (pCT) precursor proteins (ppCT50-59 and ppCT91-100) and procalcitonin (ppCT) (ppCT16-25, ppCT9-17), have been identified. These antigenic peptides based on TEIPP can effectively induce antitumor CTL effects and inhibit tumor growth [100, 101]. Therefore, targeting these TEIPP neoantigens has the potential to provide a promising new immunotherapy approach for TAP-deficient/HLA-low tumors.

### (四)、Neoantigens encoded by viral genes

Viral proteins may be considered another class of neoantigens in virus-induced tumors because they are almost completely different from normal cellular proteins and can trigger high-affinity TCR responses [102]. For example, some solid tumors are caused directly by viral infections, including cervical cancer caused by HPV virus infection [103], nasopharyngeal carcinoma caused by Epstein‒Barr virus (EBV) infection [104], and lymphoma caused by EBV infection [105]. These viral genes with carcinogenic properties can be integrated into the cell genome and promote the continuous expression of viral genes, thus leading to the occurrence and development of tumors [106, 107].

Currently, many tumor immunotherapy studies focus on virus-derived tumor antigens [108]. For example, in two of the nine patients with HPV-positive metastatic malignancies, tumor regression continued in the ACT treatment study using TILs [109]. In two separate clinical trials (NCT02280811 and NCT02858310), autologous T cells transduced with anti-E7-TCR responded in 4 of 12 patients, while T cells transduced with anti-E6-TCR responded in all 12 patients [110–112]. ACT therapy for MCPyV and EBV has also achieved promising clinical results in the treatment of corresponding tumors, although other effective therapies have been implemented in these experimental protocols. None of these clinical trial participants showed significant toxicity to normal tissue [113, 114]. Overall, these trials demonstrate the safety and efficacy of targeting oncogenic viral proteins to treat associated tumors. Given their critical role in tumorigenesis and the fact that they are shared by patients, these neoantigens are ideal targets for cancer immunotherapy, these results support the further development of integrated treatment regimens based on viral protein correlations.

### (五)、Cancer-testicular antigens

Cancer/testis antigens (CTAs) are also known as cancerous testis antigens. These antigens are not expressed in normal tissues except the testis and placenta but are abnormally expressed in a variety of tumors with high tumor specificity and strong immunogenicity [115]. More than 110 CTAs have been identified, which belong to more than ten gene families, such as the MAGE, BAGE, GAGE, SAGE, HAGE, SSX, SCP1, LAGE and NY-ESO-1 families. Among them, MAGE-A, MAGE-B, MAGE-C and NY-ESO-1 family protein molecules are more studied [116, 117]. CTA is an ideal therapeutic target of TCR-T cells. T cells targeting tumor-associated CTAs selectively eliminate tumor cells and with little or no toxicity to normal tissues [118, 119].

### (六)、Tissue differentiation antigens and others

Tissue differentiation antigens are expressed in tumor cells and some normal cells and tissues. Targeting them can cause damage to normal tissue, but these injuries tend not to be fatal [120]. The most famous of these antigens are melanoma differentiation antigens, including Melan-A, Mart-1, gp100, gp75, TRP21 and TRP22 [121]. Melanoma differentiation antigens are only expressed in melanoma and melanocytes, most of which are presented by HLA-A2 [122]. Some lung cancer antigens discovered in recent years are also tissue specific and can also be classified as tissue differentiation antigens [123]. At present, most studies on TCR-T-cell target antigens focus on polypeptide antigens that can be presented by HLA-A2. Only tumor antigens that can be recognized by T cells have been identified in several types of tumors, such as melanoma, while ideal antigens have not been found in the vast majority of tumors, resulting in less ideal tumor-specific or related antigens [124, 125]. Solid tumor cells often have low or no expression of Class HLA-I molecules and cannot effectively present their own antigens, which makes it difficult to find HLA-A2-restricted antigens. In future studies, in addition to continuing to search for Class HLA-I restricted protein antigens, Class HLA-II presented protein antigens will also be studied [126, 127]. Additionally, attention should be given to other antigens besides protein antigens, such as polysaccharide and lipid antigens [128, 129].

### 二、Identification, prediction and validation of TSAs

Thorough screening of TSAs across the entire cancer spectrum is now possible using the fusion of whole exome sequencing (WES), RNA-seq, and proteome data from TCGA. Based on NGS data, virtual peptide libraries have been created, and potential TSAs have been identified by virtual methods. The typical workflow for TSA prediction can be summarized in the following steps: mutation identification, HLA typing, screening and prioritization of neoantigens based on HLA binding affinity, and experimental validation of immunogenic neoantigens based on T-cell analysis [10] (Fig. 2d, e, f, g, h, i).

### (一)、Identification of somatic mutations

Currently, the initial phase involves mapping tumor-specific genetic abnormalities using the WES of tumor and normal tissue DNA to detect possible neoantigens from NGS data. By comparing NGS data from tumors and normal tissues in the same patient to identify genetic changes, the development of immunogenomics strategies has been greatly accelerated in terms of its ability to predict mutated peptides resulting from somatic mutations [130, 131]. WES is the recommended source of NGS data for neoantigen prediction because it provides the highest mutation coverage by focusing on the protein-coding regions of the genome [132]. RNA-seq data can be combined with WES to determine whether the mutated gene is expressed in the tumor [133]. Additional biological information can also be found in RNA-seq, such as information about copy number changes, microbial contamination, transposable elements, cell types, and neoantigen presence [134]. Mass spectrometry enables high-throughput identification of MHC-binding peptides and direct detection of MHC-binding peptides from immunoprecipitation and extraction. The neoantigens predicted by immunogenomics methods can be verified by comparing the tandem mass spectrometry of the sample with that of the synthetic peptide. Especially for rare HLA allotypes and HLA-II ligands, mapping tumor HLA ligands can help identify neoantigen-specific cancer immunotherapy targets in clinical trials [135]. MS is integrated with NGS to further detect tumor-specific neoantigens produced by somatic mutations, noncoding RNAs, and proteasome splicing, which are omitted by whole-exome- or transcriptome-based neoantigen sequencing techniques [136]. Computational analysis includes data preprocessing and quality control, identification of the variation of somatic mutations, and prediction of altered proteins and functional effects using public genome, transcriptome, and proteome databases. The immunogenicity of neoantigens, including the rank affinity of mutant and normal peptides, the frequency of mutant alleles, and the amount of gene expression, was evaluated based on the filtration technique of an integrated scoring system and quantitative scoring of the characteristics of the novel peptides to experimentally evaluate the immunogenicity of the peptides [137, 138] (Fig. 2a, b, c; Table 1).

### (二)、HLA typing

Humans have more than 24,000 distinct HLA-I (HLA-A, HLA-B, and HLA-C) and HLA-II (HLA-DR, HLA-DQ, and HLA-DP) alleles, and their mixing results in biological diversity [139]. Similar to other antigens, neoantigens are usually delivered by MHC-II molecules of CD4^+^ T cells and MHC-I molecules of CD8^+^ T cells in a cell-specific manner. The patient's HLA alleles determine their tumor-specific antigen library, which is made available to T cells for recognition. Therefore, one of the most important initial steps in neoantigen prediction is to determine a patient's HLA genotype [126]. Most methods rely on DNA-derived NGS data obtained from WES or WGS to achieve this goal. For example, Optiype and polysolver are excellent tools for identifying Class HLA-I alleles. Other types, such as HISAT-Genotype, ATHLATES, HLA-HD, HLA-Reporter, PHLAT, HLA-Scan and HLA-Profiler, can be used for HLA-I and HLA-II type classification [140–142] (Fig. 2d).

### (三)、Prediction of HLA binding and neoantigen presentation

A number of online predictive tools have been created based on the discovery of neoantigens for MHC molecular processing and presentation, including NetChop, NetCTL, and NetCTLpan. By combining HLA ligand set data into computer learning algorithms, such as linear regression and artificial neural networks, their predictive power can be actively improved [143–146]. In vitro peptide-HLA binding datasets are used to train computer learning models, and NetMHCpan and MHCflurry are the main components of the current HLA ligand recognition line. They improve the predictive performance of tumor neoantigens by integrating information from binding affinity data and MS polypeptide set data to generate "panspecific" computer learning strategies for MHC-I [147, 148]. A series of computational techniques for predicting MHC-II binding epitopes have been developed using artificial neural networks, including NetMHCII, NetMHCIIpan, SYFPEITHI, RNAKPEP, MULTIPRED2, ProPred, and MHCPred. However, current computational predictions of binding affinity for MHC-II peptides are less precise than those for MHC-I molecules. First, compared with MHC-I molecules, MHC-II binding peptides are more promiscuous in terms of peptide length and binding sequence motif. Second, the polymorphism of α and β chains in MHC-II molecules also greatly expands the diversity of peptide binding specificity [149–154]. Given that multiple processes control neoantigen presentation, it can be inferred that increasing binding affinity alone does not accurately reflect cell treatment and T-cell response. Other properties, including proteasome cleavage, transport of peptides into the endoplasmic reticulum, HLA alleles, binding affinity between peptides and MHC molecules, etc., give preference to possible neoantigens [155] (Fig. 2e, f, g).

### (四)、Evaluation and verification of the immunogenicity of candidate neoantigens

It is well known that suitable MHC molecular presentation and effective TCR recognition are prerequisites for immunogenic neoantigens. Most neoantigens predicted by MHC molecular presentation do not trigger an immune response. Therefore, it is critical to consider TCR recognition of pMHC complexes when evaluating the immunogenicity of potential neoantigens [155–157]. There are a number of programs to predict neoantigen-specific T-cell recognition, and the most common methods are NetCTL and NetCTLspan. A composite score is generated based on MHC binding, C-terminal cleavage affinity, and TAP transporter, rather than directly predicting T-cell junctions [145, 158]. Recent studies have used computer learning or deep learning techniques to predict TCR-peptide/-pMHC binding. In addition to identifying TCR-pMHC pairs, clustering methods such as pMTnet and GLIPH can also cluster TCRs that recognize the same epitopes and predict their HLA limitations [159–161]. Predicting the binding affinity of TCR remains challenging due to its low affinity for pMHC ligands (Fig. 2h). Evaluation of the immunogenicity of candidate neoantigens using a variety of screening methods will be critical to more precisely identify and select neoantigens suitable for clinical intervention [162] (Table 2). To more accurately evaluate the potential of neoantigens in immunotherapy, experimental validation of their T-cell reactivity is essential [163]. Neoantigen reactive T cells have been validated or screened by T-cell-based assays, multicolor labeled MHC tetracers, and enzyme-linked immunosorbent spot assays (ELISPOTs).

**Table 2:** Immunological screening assays used to test for neoantigen recognition

Strategy	Advantages	Disadvantages
cDNA libraries	Interrogates all transcribed sequences	Interrogates mutated and non-mutated sequences; Biased toward highly transcribed genes; Influenced by the size, expression levels or GC-richness of transcripts encoding for T-cell epitopes; Labor intensive and time-consuming
Peptide-HLA multimers	Allows the isolation of antigen-specific T cells; Overcomes the need of autologous APCs	Multimers are available for a limited number of HLA molecules; Exclusively interrogates a selected list of mutated epitopes based on in silico prediction or validated by immunopeptidomics; Not optimal for CD4^+^ cells
Minimal epitopes	HLA-matched target cells (based on in silico prediction) can be used instead of autologous APCs; Cost-effective	Requires autologous or HLA-matched cells as target cells; Exclusively interrogates a selected list of mutated epitopes based on in silico prediction or validated by immunopeptidomics; Not optimal for CD4^+^ cells
Tandem minigenes or peptide pools	Allows potential processing and presentation of candidate neoantigens on HLA-I and HLA-II; Does not require prior knowledge of the minimal epitope or HLA restriction; Can be used to interrogate all or a large portion of mutated epitopes;	Peptide processing by immunoproteasome in APCs might differ from processing by the proteasome in tumor cells; Availability of APCs/effectors can limit this approach, especially when > 250 epitopes are tested; Cost increases in patients with high mutation burden; Requires autologous APCs as target cells;

*APC* Antigen-presenting cell, *HLA* Human leukocyte antigen

#### Validation of the immunogenicity of neoantigen peptides

The specimens used for transplantation of PDTX (Patient-Derived Tumour Xenografts) in humanized mice are directly derived from human tumor tissues and have not been cultured in vitro, stably retaining the genetic, histological, and phenotypic characteristics of the tumor, i.e., tumor heterogeneity [164]. Therefore, PDTX can be used to screen the sensitivity or resistance of immune cells by MHC tetramers, and the test results have good clinical predictability. In addition, because PDTX better retains the tumor mesenchymal and stem cell components during the transplantation process, the microenvironment for tumor growth is closer to the actual situation. PDTX can also provide a large number of specimens for the preservation and passage of tumor samples, which can be used before clinical treatment for the efficacy validation of the immunogenicity of cells or vaccine preparations. It can also be used to determine the appropriate sample size and efficacy evaluation for small preclinical trials. The PDTX test can be used to predict and validate the efficacy of neoantigen in advance, improving the effectiveness rate of personalized tumor neoantigen therapy [165–167].

#### In vitro validation of immunogenicity by ELISPOT test

The T-cell immunogenicity assay is the most direct method to evaluate the immunogenicity of candidate neoantigens. The reactivity of neoantigen-specific T cells amplified in vitro was measured by flow cytometry for the T-cell activation markers 4-1BB and OX-40 after polypeptide stimulation or by ELISPOT for IFN-γ production. In organisms, cells will produce cytokines (such as IFN-γ) after stimulation. These cytokines will be captured by specific antibodies and then develop colors to form spots. The number of spots represents the level of immunogenicity of the corresponding neoantigen. This platform will further enhance the accuracy of neoantigen screening at YuceNeo. ELISPOT has little effect on the biological process of cells, and higher sensitivity in cell detection. Based on test on the single-cell level, one IFN-γ-secreting cell can be detected from 200,000 to 300,000 cells through the ELISPOT test. ELISPOT is more sensitive than Elisa and the limiting dilution method [168–172].

#### In vitro validation of immunogenicity by NEST

In addition, scRNA-seq was used to discover paired TCR sequences associated with cells expressing high levels of IFN-γ in TILs that were cocultured with tandem microgene (TMG)-transfected or peptide-stimulated antigen-presenting cells (APCs) [173]. ELISPOT can be used in vitro to analyze which neoantigens can stimulate T cells to secrete cytokines, but it cannot detect the TCR of tumor-specific T cells. YuceNeo's unique NEST (Neoantigen Expansion of Specific T Cells) validation technology integrates TCR sequencing of peptide-stimulated T cell culture with a biological information platform to identify antigen-specific clonal amplification. The NEST platform of YuceNeo compares the TCR databases with and without stimulation by neoantigen peptides. If a TCR with an increasing frequency can be found, it can indicate that the corresponding neoantigen is immunogenic. The NEST technology platform not only can validate the immunogenicity of neoantigens screened by TruNeo™ in a high-throughput manner, but also can be used to dynamically monitor the effect of tumor immunotherapy [174–176]. Based on WES-guided neoantigen prediction and TCR sequencing of polypeptide-stimulated T-cell cultures, neoantigen-specific clones of TCRVβ were sensitively characterized by the mutation-associated neoantigen functional expansion of specific T cells (MANAFEST) assay [177, 178]. In addition to evaluating the tumor specificity of the TCRVβ clones, dynamic changes in neoantigen-specific T-cell responses over time can be studied, and the effects of immunotherapy can be monitored using liquid biopsies obtained before or after treatment [179, 180] (Fig. 2i).

### 三、Enrichment and isolation of tumor antigen-specific T cells

V(D)J recombination of TCR during thymus development leads to great diversity in TCR sequences in the human T-cell pool. It is estimated that in the average adult, there are approximately 4 × 10^11^ total circulating T cells and approximately 10^10^ unique T-cell clones. Therefore, T-cell clones are specific for the vast majority of nonviral antigens. The frequency of cloning in peripheral blood is much lower than the frequency of isolation of antigen-specific TCRs under current technical conditions [181–183]. Thus, TCR isolation efforts usually begin with methods that allow enrichment of T cells with the desired antigen specificity (Fig. 2i).

### (一)、Enrichment of antigen-specific T cells

#### Amplification of TILs

A large number of tumor-infiltrating lymphocytes (TILs) are usually present in some types of solid cancer. Compared with peripheral blood T cells, T cells in tumor tissues are usually enriched in tumor antigen-specific clones [184–186]. Some research groups have used extended TILs as a source for finding tumor-specific TCRs [175, 187]. In addition, TIL amplification in vitro is itself an effective adoptive T-cell (ATC) therapy for several types of solid cancer [188, 189]. Studies have shown that Iovance's TIL treatment named Lifileucel has strong efficacy in clinical trials [190, 191].

#### Vaccination

T cells that are specific to the antigen of interest can be selectively expanded in vivo through vaccination strategies. A common approach is to inoculate human HLA transgenic mice with antigens of interest, which results in robust enrichment of antigen-specific T cells that can be harvested from the lymph nodes and spleen [192, 193]. In certain cases, peripheral blood from patients participating in cancer vaccine trials has been used as a source of antigen-rich T cells for TCR [194].

#### Selective in vitro expansion of peripheral blood T cells

Peripheral blood T cells were stimulated in vitro in an antigen-specific manner to drive selective T-cell expansion with the desired specificity. Early pioneering work in this area included in vitro stimulation of peripheral blood T cells to preferentially expand virus-specific T cells [195, 196]. These stimulation methods have been used to amplify TAA-rich and neoantigen-specific T cells [197, 198]. These methods usually stimulate T cells by autologous antigen presenting cells (APCs), usually dendritic cells (DCs), in the form of exogenous peptides or by cDNA/RNA delivery pulsed with the antigen of interest [198–202]. Several studies have shown that in the case of patient-derived peripheral blood, initial selection of PD-1^+^ and/or antigenic experience (CD45RO^+^CD62L^+^, CD45RO^+^CD62L^−^ or CD45RO^−^CD62L^−^) T cells can further enhance tumor-specific T-cell enrichment in vitro [203, 204]. To overcome the need to produce autologous mature DCs for antigenic stimulation, so-called artificial antigen presenting cells (aAPCs) have been developed [205]. For example, the myelogenous leukemia cell line K562, a common aAPC system, was used, and this cell line was negative for HLA-A, B and DR. This cell line acts as a modular aAPC by providing the stable transduction of various HLA alleles and costimulatory molecules [206]. Other cell-free aAPC systems have also been developed to conjugate HLA and costimulatory molecules to beads and nanoparticles to stimulate peripheral blood T cells and obtain a large number of clinically needed antigen-specific T cells [207, 208].

### (二)、Isolation of antigen-specific T cells

After obtaining polyclonal T-cell products enriched with target-specific T cells, it is necessary to isolate antigen-specific T cells from a large population of T cells. Approaches in this area usually involve stimulating T cells with homologous antigens of interest and isolating antigen-reactive T cells based on the increased expression of molecules known to be associated with T-cell activation [209]. For example, 4-1BB and OX40 in CD8^+^ T and CD4^+^ T cells allow for the isolation of these cells by FACS sorting or magnetic bead sorting [210]. Alternatively, the use of peptide-HLA polymeric MHC staining followed by FACS sorting or magnetic bead sorting is an effective way to identify and isolate antigen-specific T cells. Although the library of HLA polymeric reagents is expanding, these reagents are still limited to relatively common HLA alleles [170]. Another approach is IFN-γ capture; IFN-γ is rapidly secreted by antigen-stimulated CD8^+^ T and CD4^+^ Th1 cells, thus, IFN-γ capture identifies and captures antigen-stimulated T cells based on IFN-γ production [211, 212].

### 四、TCR epitope discovery strategy: from TCR to epitope

#### (一)、Prediction strategy based on computer algorithms

The binding affinity of HLA and the immunogenicity should be considered in the prediction of antigen epitopes [213]. Antigenic peptides can be run through MHC epitope prediction algorithms to prioritize the list of candidate antigens. The following relevant methods have been used to develop relevant prediction software: structure-based (SB), motif matrices (MM), quantitative affinity matrices, QAM), artificial neural network (ANN), support vector machines (SVM) and so on. The prediction algorithms include NetMHC-4.0, Immune Epitope Database (IEDB), NetMHCpan-4.1, NetMHCIIpan-4.0, SYFPEITHI and HLAthena, which are used to calculate and predict TCR epitopes [150, 214–217].

#### (二)、Screening strategies based on tumor cells

HLA peptides were obtained by an immunoprecipitation technique, and the products were acid-eluted to obtain peptides; then, the peptides were identified by mass spectrometry. The quality of peptide segments was determined by primary mass spectrometry, and peptides with high abundance were selected for secondary mass spectrometry. In secondary mass spectrometry, the peptides collided with each other, resulting in amino acid bond breakage, and peptide fragment ions were analyzed by the detector to obtain the amino acid sequence information of screened peptide segments [218, 219]. The technical route is relatively mature, and low-abundance epitopes are difficult to identify.

#### (三)、T-cell-based screening strategies

##### T-Scan

The new method of high-throughput, concentrated screening and recognition of T lymphocyte target antigens in the whole genome can be used to identify antigens that can be effectively recognized by T cells. The human whole genome target antigen library was transduced into target cells containing the granzyme reporting system. After coincubation with T cells expressing TCR, the target antigen of the TCR could be enriched and identified. T-scans can be used to recognize functional target antigens of CD8^+^ T cells and can repeatedly detect target antigens from a large number of memory T cells [171, 220]. They have the advantages of genome-wide identification of target antigens, T-cell-based functional tests, and identification of potential off-target antigens.

##### Cellular system

After transduction of the target antigen library into target cells containing the Trogocytosis reporting system and coincubation with T cells expressing TCR, the target antigen of TCR can be enriched and identified. Nibbling is highly specific and occurs only between cells successfully recognized by TCR antigens. TCR-Jurkat T cells expressing the known pairing were injected into a mixture of APC cells with the specific pMHC molecule recognized by the cells at a ratio of 1/10000. By flow cytometry, nibbling was still able to accurately label more than 70% of the APC cells specifically paired with this TCR, while other unrecognized antigen-presenting cells were not [221, 222]. This strategy also has the advantage of genome-wide identification of target antigens, T-cell-based functional tests, and identification of potential off-target antigens.

##### SABAs system

The target antigen library was transduced into target cells containing the TCR-PMHC bifunctional molecule reporting system, and the target antigen of the TCR could be enriched and identified after coincubation with T cells expressing the TCR [223, 224]. Advantages include genome-wide identification of target antigens, T-cell-based functional tests, and identification of potential off-target antigens.

##### Presenter system

After the target cells expressing the antigen library were coincubated with T cells expressing the TCR, the target cells that were killed in significant amounts were identified by high-throughput sequencing to identify the target antigen of the TCR [225].

##### RootPath system

After the TCR sequence was found by single-cell sequencing, the quantized TCR was synthesized. After coculture with target cells expressing Mini-Gene, tumor reactivity TCR was confirmed by a T-cell reporting system. The T cells carrying this TCR were then assessed to determine if they could recognize the tumor and react with it [110, 226]. This system has the advantages of quantized TCR synthesis and T-cell functional tests.

##### MHTEM system

Libraries of multiple HLAs and the target antigen MiniGene were introduced into target cells. After coculture with T cells, the target cells that were killed in significant amounts were identified by high-throughput sequencing to identify the target antigen of this TCR [227, 228]. The advantages of this system are based on T-cell functional testing, simultaneous screening of multiple HLA presentation epitopes and identification of potential off-target antigens.

### (四)、Strategy based on yeast display

Exogenous target protein gene sequences (exogenous proteins) were fused with specific carrier gene sequences and introduced into yeast cells. The yeast intracellular protein transport mechanism (GPI anchoring) was used to express target proteins and locate them on the yeast cell surface. Yeast was used to display random amino acid epitopes, and the expression of TCR protein was used to enrich potential antigenic epitopes; this system is also known as the 3T system [229, 230].

### 五、TCR cloning strategies: from known epitopes to specific TCRs

**Fig. 3:**
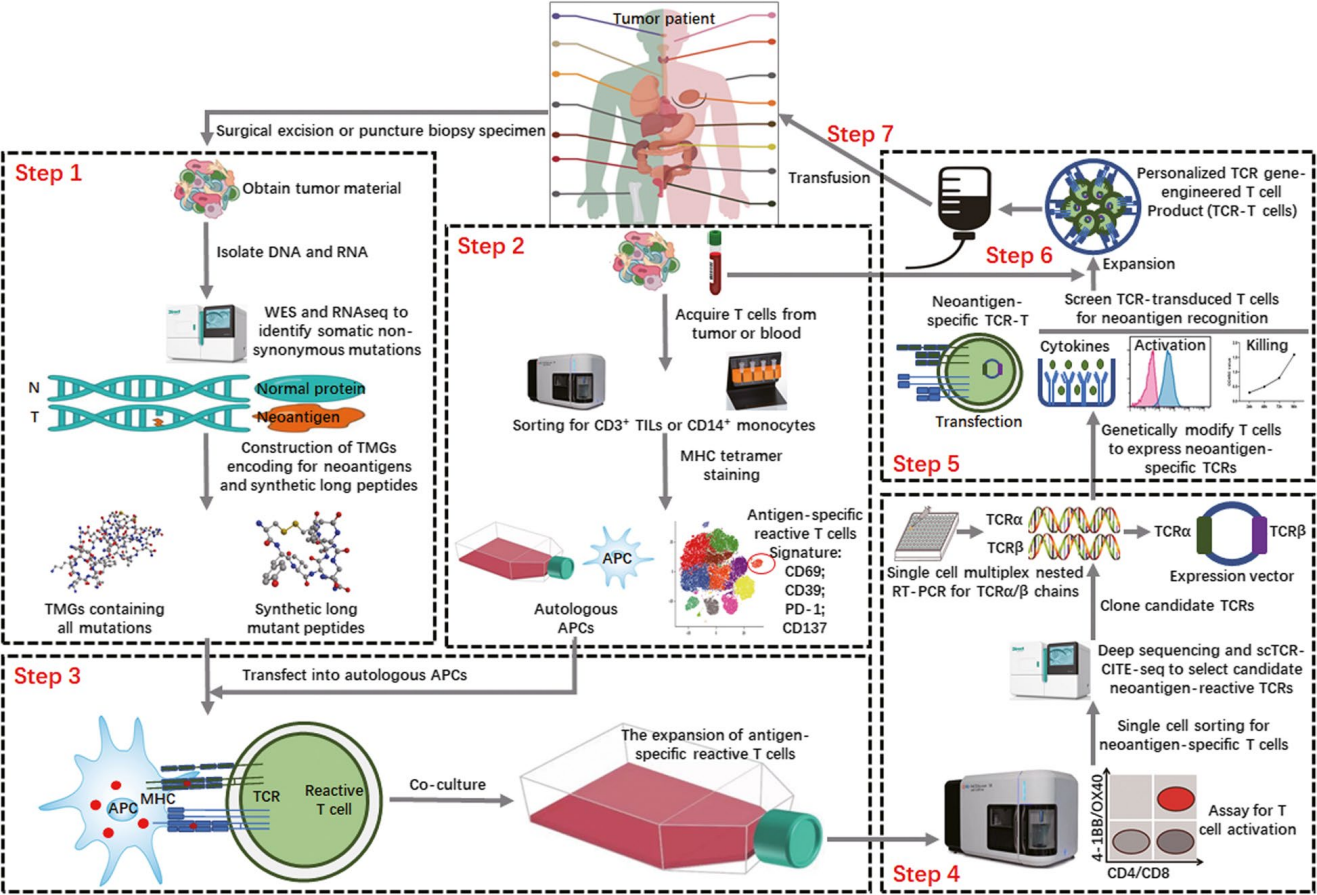
Rapid identification of neoantigen-specific TCRs for personalized engineered TCR-T-cell therapy. Tumor (T) and normal (N) DNA are used to conduct WES and RNA-seq to identify cancer-specific nonsynonymous mutations. Candidate neoantigens are used to design tandem minigenes (TMGs) encoding mutant peptides and synthesize mutant peptide libraries (Step 1). TILs and PBMCs are isolated from single-cell suspensions taken from patient samples. TILs and PBMCs are analyzed using single-cell TCR-CITE-Seq, and a combined tag of gene and surface protein expression is used to predict candidate neoantigen-reactive T cells (Step 2). Candidate antigen-reactive T cells are cocultured with autologous antigen-presenting cells (APCs) expressing candidate neoantigens (TMG or peptides), and antigen-specific T cells are amplified (Step 3). Antigen-specific T cells are selected by flow cytometry, and neoantigen-reactive TCRs are identified and screened by scTCR-CITE-seq or deep sequencing. Then, the TCRα/β chain is reverse transcribed by single-cell multiplex nested RT-PCR, and its related plasmid is constructed (Step 4). T cells expressing candidate reactive TCRs are generated by cloning the selected TCR sequence into a retrovirus vector and transducing T cells. The recognition of neoantigens by T cells transduced by the TCRα/β chain is verified by different screening experiments (Step 5). PBMCs are obtained from patients, and the reactive T cells of the neoantigen-specific TCRα/β chain are amplified by the above methods (Step 6). Validated neoantigen reactive TCRα/β chains are selected to design final personalized TCR-engineered T (TCR-T) cell products that will be injected into patients for cell therapy (Step 7)

The specificity of TCRs is determined by the regional coding of two separate genes, TCRα and TCRβ, and presents unique challenges in determining functional TCR sequences from T-cell populations. Accurate sorting of TCRs is the key to designing TCR-T-cell therapy for subsequent treatment [231–233] (Fig. 3).

### (一)、Strategies based on tetramer staining

#### Multicellular TCR cloning

Multiple cells stained with tetramers positive for specific antigens were selected, and the TCR sequences of these positive cells were amplified. The method in not technically difficult and has a high success rate of amplification. However, a large number of cross-pairing experiments are needed to verify the pairing and specificity of TCR chains, and important TCR chains may be missed [231, 234].

#### Single cell TCR cloning

Individual cells stained with tetramers positive for specific antigens were selected, and TCR sequences were amplified. Some studies have amplified the TCR-α and -β chains by performing single-cell RT‒PCR, thereby avoiding the need to amplify T-cell clones after antigen-specific T-cell isolation. In this method, a single T-cell was sorted by FACS into a well containing RT‒PCR buffer, RT‒PCR was performed from the single cell, and the TCR-α and -β chains were amplified by PCR. This approach reduces the time and labor required to amplify a single T-cell clone; however, the disadvantage of this approach is that confirmatory assays to assess antigen specificity cannot be performed on T-cell clones prior to sequencing [235, 236]. Single-cell RNA sequencing (scRNAseq) has also emerged as a unique and effective platform for TCR discovery that allows for single-cell evaluation of cellular gene expression and gene transcription. As a result, several recent studies have successfully used the platform for TCR discovery. The method involved stimulation of T cells with the antigen of interest and then conducting scRNAseq sequencing analysis. Antigen-specific T cells are identified by effector cytokines such as IFN-γ, TNF-α, and/or IL-2. Transcripts of the TCR-α and -β chains of activated cells were then obtained from the same dataset [175, 237]. This method has the advantages of low cost, high speed and high specificity and can directly realize TCR pairing, but it involves relatively complicated technology.

#### Feature barcoding technology

Tetramer-stained cells were prepared by single-cell droplets, and cell membrane DNA tags, RNA expression and TCR-V(D)J libraries were constructed and sequenced. 10 × Feature barcoding technology was used to construct a library by magnetic bead enrichment of tag sequences containing cell surface proteins and antigen-MHC complexes. The relatively long portion is then used to construct an mRNA library. A V(D)J library was constructed by enrichment of the V(D)J region by nested PCR [238, 239]. This method can simultaneously clone the specific TCR of multiple epitopes. However, the disadvantages include the fact that there are limited cell fluxes that can be analyzed, the large amounts of TCR synthesis needed, the limited sources of T cells, high cost and long cycles.

#### HLA-peptide-cDNA tag complex technology

DNA tags were used to label epitopes with multiple specificities, and tetramers were used to stain multiple epitope-specific T cells simultaneously [240, 241]. This method can be used to simultaneously clone the specific TCRs of multiple antigen epitopes, but it is difficult, and nonspecific reactions are problematic.

### (二)、Strategies based on T-cell functional analysis

#### Single-cell RNA + VDJ sequencing

The antigen-stimulated T cells were sequenced by single-cell RNA + VDJ sequencing using 10 × RNA expression + TCR VDJ sequencing, independent of tetramer [242, 243]. However, non-specificity increases the subsequent workload, cost and cycle time. Normal T-cell activity is needed, and TILs with poor activity are not appropriate.

#### Activated marker sorting

Activating molecules (PD1, 4-1BB) were used to sort antigen-activated T cells independently of tetramers [244–246]. This method was dependent on normal T-cell activity and not applicable for TILs with poor activity.

#### Intracellular factor sorting

T cells were stained with intracellular factors after antigen stimulation, independent of tetramers [247]. This technique relies on normal T-cell activity, and TILs with poor activity are not suitable. The operation is complex and technically difficult.

#### Lightning optofluidic system

Berkeley Lights' Lightning optofluidic platform enables researchers to precisely study the behavior of single cells within a specified time frame by recording video of the cells throughout the data collection process. The platform works through the microfluidics of a silicon chip the size of a postage stamp that contains tiny nanopens and long, narrow chambers for isolating and growing individual cells. This system can be used to gain unparalleled biological insight. Lightning's new optofluidic technology can isolate and characterize 1,000 single cells on a single platform. Single-cell analysis has traditionally required multiple instruments over a period of several months. The Lightning system allows cells to clone and grow and be analyzede and recovered in a matter of days. After automatic cloning, the cells were individually detected and monitored using bright field and fluorescence techniques. This allows for the creation a visual record of every cell in every experiment, and the understanding of this complex biology at a level that cannot be achieved by any other method. In immunogenomics-related research, the system can be used to selectively derive target T cells/single T-cell clones after analysis and evaluate them for subsequent downstream sequencing and other experiments. Research at the single-cell level and rapid and efficient construction of CAR/TCR vectors have accelerated the process of T-cell genetic modification [248, 249].

#### Droplet entrapping microfluidic (DRRM) cell system

This system is an ultrahigh throughput single cell sorting platform developed based on droplet microfluidic technology. By using the latest microfluidic technology, thousands of microdroplets can form every second. Cells are encapsulated in microdroplets and can undergo biochemical processes such as growth, cleavage, metabolism and reaction. Fluorescence signals of different intensities are produced when the cell fully combines with the fluorescent sieve in the droplet. The low- and high-yield cells were separated by fluorescence signals using microdroplet separation technology for high-pass quantification of the separation process. Activated T cells were detected and analyzed by a microfluidic system, and the TCR was cloned. The technique is based on T-cell functional analysis, which reduces nonspecific findings [250, 251]. However, the number of T cells in each analysis was limited, and the system complexity was high.

### (三)、High throughput library strategy

High-throughput human-derived TCR libraries generate single-cell droplets from a large number of patient-derived tumor infiltrating lymphocytes (TILs). TCR-α and -β were amplified in the droplet, and the TCR-α and -β chains were fused in series. TCRs were expressed in series in the vector, TCR plasmid and virus library were formed, and the human TCR library was constructed. After stimulation with the T-cell reporter system and specific antigen epitopes, high-throughput screening of specific TCRs was performed to clone antigen epitopes directly from the TCR library [252–254]. This method is based on the T-cell function of TCR discovery and the specificity is good;, this method allows for selection of a specific affinity interval of TCR. The constructed library resources can be regenerated and gradually eliminate TIL resource dependence, but the technical difficulty is high.

### 六、TCR-T cells based on specific TCRs

Adoptive T-cell therapy (ACT) uses the patient's own naturally occurring or genetically engineered antitumor lymphocytes, so neoantigens with high immunogenicity provide an excellent target for ACT therapy [255]. Currently, different ACT technologies are being developed, including tumor-infiltrating lymphocytes (TIL), chimeric antigen receptor T (CAR) cells, and T-cell receptor engineered T cells (TCR-T), which have been successfully used to treat a variety of malignancies [256, 257]. However, the clinical efficacy of TIL and CAR T cells in solid tumors is not ideal due to a number of obstacles, including scarcity of available antigens, tumor heterogeneity, or tumor immunosuppression. Advanced solid tumors are also characterized by connective tissue hyperplasia and abnormal angiogenesis, leading to hypoxia and altered nutrient supply, both of which contribute to poor clinical outcomes [258, 259]. TCR-T-cell therapy is an alternative with several advantages. First, the target antigen pool of TCR-T cells is larger than that of CAR T cells. Second, the epitope density required by TCR-T cells to induce activation is lower than that of classic CAR T cells (1–50 vs. 10^3^ epitopes per cell). This increased sensitivity may lead to an improvement in the detection and lethality of tumor cells. Finally, the high affinity of TCR-T cells may also enhance their efficacy. The affinity of TCR-T cells for target cells was lower than that of CAR T cells. It is possible to allow each TCR-T-cell to "scan" and clear several antigen-presenting tumor cells [176, 260] (Fig. 3).

### (一)、Definition of TCR-T cells

TCR is a receptor expressed on the surface of T cells that can specifically recognize related antigens expressed on the surface of tumor cells and presented by MHC, thus mediating antitumor effects. In tumor patients, tumor reactive T cells are present. However, these natural antitumor cells cannot be used for clinical treatment due to their limited source, difficulty in isolation, and difficulty in large-scale expansion in vitro. To overcome these difficulties, TCR-coding genes can be isolated from tumor-responsive T cells, introduced into ordinary T cells and imbued with tumor-specific killing capabilities. In this way, a large number of antigen-specific T cells can be rapidly produced to meet application requirements. These T cells that express exogenous TCR and recognize specific epitopes are called TCR-T cells [176, 261]. The main difference from CAR T cells is that only TCR sequences (α and β chains) are introduced into TCR-T cells. TCR signal transduction depends on the host’s T-cell signaling pathway. In addition to single-chain variable fragment (scFV) sequences containing specific antigens, CAR molecular sequences also contain functional motifs of costimulatory signaling molecules (CD28 and/or 4-1BB). TCR-transduced T cells can target any surface or intracellular antigen [262]. Several clinical trials have demonstrated the feasibility of effective methods for the identification of new antigens for the design of specific TCR-T cells. When neoantigens are recognized and predicted, new epitope-specific T cells are isolated, and their TCRs are sequenced. Candidate TCR sequences with known neoantigen reactivity can be introduced into T cells by transposon or CRISPR/Cas9 systems. These engineered cells expressing neoantigen-specific TCR (TCR-T) were injected into patients to determine their antitumor effects after their tumor activity was validated [111, 263–265].

### (二)、Selection of therapeutic target antigens for TCR-T cells

TCR-T cells recognize tumor cells based on the recognition of pMHC/peptides after antigen processing. Thus, antigen specificity determines the accuracy of TCR-T-cell killing [266, 267]. The ideal tumor antigen should be selectively expressed in tumor tissue and not expressed in normal tissue to avoid causing an autoimmune response. At the same time, the targeted antigen should be immunogenic to induce an effective antitumor immune response. The ideal target antigen of TCR-T cells is TSA. TSA is mainly the product of gene mutation. This type of antigen is only present in tumor cells but is not expressed in normal cells and tissues. Theoretically, TSA is an ideal target for TCR-T-cell therapy. The tumor specificity of TSA means that there is no autoimmune tolerance and that the immune response to TSA does not damage normal tissue. However, there are some problems in the study of TCR-targeting TSA. On the one hand, the immunogenicity of antigenic peptides produced by gene mutation is relatively weak, and it is difficult to isolate TCRs with high affinity. On the other hand, TSA is often specific to the individual tumor or even present only at certain points in time. Targeting such antigens requires a very individualized TCR-T-cell preparation process [10, 268–272]. At present, it is necessary to actively search for tumor-specific neoantigens with strong immunogenicity and carry out individualized clinical treatment of TCR-T cells.

### (三)、Antigen presentation by TCR-T cells

The prerequisite for TCR-T cells to recognize target cells is the presence of antigen epitopes on the surface of target cells, which involves antigen presentation [176]. According to the origin, processing method and MHC, antigen processing and presentation can be completed in the cytosol and lysosome, respectively. The processing and presentation of endogenous antigens occurs as follows: the endogenous synthesis of protein molecules includes endogenous antigens, which are also the main targets of TCR-T cells for tumor therapy. The degradation process of endogenous antigens in cells is not fundamentally different from that of ordinary proteins. In fact, endogenous antigen degradation in the cytoplasm utilizes the degradation mechanism of normal intracellular protein conversion. Endogenous antigens are degraded into 5 ~ 15 amino acid peptides by proteases in the cytoplasm, which are processed and presented by the cytosol pathway and restricted by MHC-I molecules. Exogenous antigens are degraded in endosomal lysosomes to produce peptides, some of which are 13–18 or even 0 amino acids in length and can bind to appropriate MHC-II molecules. is the antigens are processed and presented through the lysosome pathway and dependent on MHC-II for delivery to the cell surface. Dendritic cells and macrophages are the main APCs involved in the nonclassical pathway [127, 155, 156, 273]. Only antigens that can be presented are likely to be recognized by T cells. There are MHC differences among different individuals. MHC mismatches between the input cell and the recipient results in immune rejection. As a result, TCR-T cells are mainly transfused after somatic modification. To some extent, the application of TCR-T-cell therapy is limited. T cells recognize target cells mainly through the recognition of specific pMHCs by TCRs. TCR gene rearrangement can lead to approximately 10^18^ TCR sequences, which makes it difficult to identify and isolate specific TCR sequences. However, due to the strict conformational requirements for the binding of peptides and MHC, the same TCR can recognize the same pMHC in different individuals, which also lays the foundation for the universality of TCRs [126, 209, 274]. Therefore, the T-cell memory response is constrained by both antigen specificity and MHC allele specificity.

### (四)、The therapeutic process of TCR-T cells

TCRs recognize pMHC/peptides to mediate the recognition and killing of tumors by T cells [176]. A large number of antigen-specific T cells can be generated rapidly by isolating the α and β chains of TCRα and β that are responsive to tumor antigens and transferring them into T cells in a viral or nonvirus-mediated manner. The specific antigen reactive TCR sequence can be isolated, stored and amplified in plasmid form and can be used immediately when needed. This makes the threshold for clinical application of TCR-T cells much lower. The TCR gene can be used as a ready-made reagent for the treatment of tumor patients expressing specific antigens and MHC-restricted molecules [260, 261]. Therefore, the following general process of TCR-T-cell therapy for tumor patients is shown in the figure: ① Peripheral blood was extracted from patients, and T cells were isolated; ② T cells were activated by adding CD3/CD28 beads and IL-2; ③ TCR genes were transferred into activated T cells by virus or other means; ④ T cells (TCR-T cells) with specific TCR transduction were further cultured and expanded to a suitable number; ⑤ Before infusion of TCR-T cells, patients were given moderate chemotherapy or radiotherapy to remove lymphocytes from the body and enhance the therapeutic effect of TCR-T cells; ⑥ A sufficient number of TCR-T cells were injected intravenously into the patient; and ⑦ The patients were closely observed after transfusion, and the clinical efficacy and related side effects were evaluated later [176, 226].

### (五)、Toxicity evaluation and efficacy monitoring of TCR-T-cell therapy

Assessment of the toxicity associated with TCR-T cells included bioinformatics, immunological analyses of transcription and proteome databases, and in vitro tests assessing the ability of TCR-T cells to recognize normal cells or tissues. Off-target toxicity or cross-reaction is associated with TCR recognition of antigens on normal cells that are different from those that are targeted. Therefore, it is necessary to include a strategy for evaluating TCR cross-reactivity at the preclinical level, especially when the TCR sequence has been modified to improve affinity. Reliable immune surveillance is essential to assess whether neoantigen-based immunotherapy can be used to achieve the desired immune effect and to extend the list of effective immune candidates to include larger and suitable patient subpopulations. Methods to predict off-target and off-tumor toxicity (presence of cross-reactivity epitopes in normal tissues) includes the following: In silico prediction, which includes Motif blast, and HLA-binding prediction and expression; structural modeling of the pMHC interface; and TCR clustering. Experimental evaluation includes the following methods: alanine/glycine scan, combination peptide libraries (X-scan) and barcoded peptide libraries; in vitro cytotoxicity testing against primary healthy cells; and TCR-based histological analysis [275–279]. Tumor-reactive T-cell responses are critical to the antitumor effectiveness of various therapies, including cancer vaccines, ACTs, bsAbs, and ICBs. To predict the effect of cancer immunotherapy, the number and quality of tumor-reactive T cells can be measured and tracked. A number of effective markers, including CD39, PD-1, TIM-3, OX40, 4-1BB, IFN-γ, and TNF-α, can be used to determine the proportion of neoantigen-reactive T cells in infusion products and their ability to recognize autologous tumors. CCR5 and CXCL13 can also be used as T-cell intrinsic indicators of CPI sensitivity [244, 280–287]. It should be noted that circulating tumor DNA (ctDNA/cfDNA) levels, as a proxy for tumor load, can be used to dynamically detect neoantigen-producing mutations [288–291]. The characterization of neoantigen-specific TCR clonal types characterizes TILs in tumors, allowing for prediction of TCR based solely on TIL transcription status in neoantigen-specific cancer immunotherapy. These characteristics can provide a degree of clonability. Prediction of the clinical response to various immunotherapies can be performed by identifying anticancer TCRs in the blood, and more critically, without the need for functional screening of putative neoantigens in the tumor.

### (六)、Enhanced durability and antitumor function of TCR-T-cell therapy

T-cell persistence is a basic requirement for sustained immunosurveillance. Many clinical trials have shown that most unresponsive patients have no persistence of tumor-specific T cells transfused in vivo. In contrast, patient-engineered T cells that achieved a complete response or had no recurrence and tumor control demonstrated strong proliferative capacity and long-term persistence [168, 292]. To maintain T-cell persistence, multiple cytokines are used in combination to support T-cell survival and expansion. Systemic delivery of IL-2 is known to amplify T cells while maintaining functional activity. Long-lasting regression has been achieved in some patients with metastatic melanoma and kidney cancer and has been approved by the FDA. This strategy is currently used in CAR T and TCR-T-cell immunotherapy [293–296]. IL-7 is a hematopoietic cytokine that regulates many aspects of T-cell biology and is essential for T-cell survival, homeostasis, and proliferation. It promotes the survival of naïve and memory T cells by upregulating the expression of the anti-apoptotic molecule Bcl-2 [297, 298]. IL-12 is a major contributor to effective antitumor immune responses. It activates the effector function of T cells by inducing cytotoxic enzymes such as perforin and cytokines [299, 300]. IL-18 is another cytokine that has the same biological effect as IL-12 but is less toxic [301, 302]. Studies exploring the substitution of IL-18 for IL-12 have shown that CAR T cells designed to secrete IL-18 improve the survival and antitumor activity of CAR T cells in vitro and in vivo by producing IFN-γ and several other cytokines. IL-18 stimulates the expansion of human CD4 cells and activates the endogenous immune system of well-immunized mice [303, 304]. IL-15 is known to stimulate stem cell memory T-cell (Tscm) production, with the potential to maintain long-lasting T-cell responses [305]. IL-7 and IL-15 induce the generation of human memory stem T cells from naive precursors [306]. Unlike IL-2, IL-15 does not bind to the IL-2Rα chain and therefore does not stimulate Tregs and may have a more selective effect. Compared with IL-2, IL-15 tends to enhance the antitumor activity of CAR T cells by preserving their Tscm phenotype [307, 308]. IL-21 is a newly discovered member of the common gamma chain cytokine family. Similar to IL-12 and IL-15, IL-21 does not stimulate Tregs. In contrast, it inhibits the amplification of Tregs by inhibiting Foxp3, thus favoring the enrichment of antigen-specific CD8^+^ T cells [309, 310]. IL-21 promotes the maturation and enhanced killing activity of CD8^+^ T cells and promotes the differentiation of memory CD8^+^ T cells. IL-21 promotes antigen-specific CD8^+^ CTL production in vitro and performs much better than IL-2 or IL-15 in vivo in mouse models [311, 312]. These results suggest that cytokines can be used alone or in combination with other cytokines to produce tumor-specific T cells with a memory phenotype. Cytokines enhance the persistence, proliferative ability and antitumor effect of adoptive cancer immunotherapy.

### (七)、Factors influencing TCR-T-cell therapy

Studies have shown that TCR-T cells can produce a large number of inflammatory factors, such as IFN-γ, IL-2 and TNF-α, when they encounter antigen-positive tumor cells. They exhibit antigen-specific cytotoxic effects and induce antigen-stimulated reactive proliferation [261]. With the development of molecular biology, genetic modification methods and transduction technology, the construction system of TCR-T cells has made great progress, which lays a foundation for the further improvement of the therapeutic effect of TCR-T cells. As with other ACT techniques, TCR-T-cell therapy has the following advantages: ① the production of a large number of tumor antigen-specific T cells for treatment in a short period of time; ② T cells can be treated and amplified in vitro to bypass the effects of immune dysfunction in patients, and create a large number of effector cells; ③ the activation and expansion of T cells in vitro prevents the serious side effects caused by the application of biological agents in patients; and ④ by the in vitro culture of T cells, T cells can be differentiated by adding different cytokines or reagents to obtain more powerful TCR-T cells with a more lasting killing effect [226, 260, 268, 313]. However, the therapeutic effect of TCR-T cells, similar to other ACTs, is affected by a variety of factors.

#### Number of cells transfused

The ratio of TCR-T cells to tumor cells is directly related to the therapeutic effect [261]. Studies have shown that effector cells should account for at least 1%-10% of total T cells. In other words, the number of effector cells injected into the human body should reach (2–20) × 10^9^ to effectively control the tumor. However, inputting too many effector cells at a time can lead to serious off-target effects and even death. Therefore, the number of specific T cells in patients can account for 5% of total CD8^+^ T cells through repeated transfusions of a small number of cells each time. Animal experiments have shown that repeated cell inputs can help maintain the continuous attack on tumor cells and play a role in gradually shrinking the tumor [111, 176, 314, 315].

#### Immunosuppressive factors

When effector cells are imported back into the body, they are inevitably affected by the immunosuppressive state of the body. In early studies, to enhance the activity of transfused cells, the main method was to administer IL-2, IFN-γ and other cytokines simultaneously. However, while enhancing the activity of transfused cells, IL-2 also promotes the expansion of CD4^+^CD25^+^ Treg cells, which are an important class of immunosuppressive cells [293, 316, 317]. In addition, immune checkpoints have been found to be one of the important factors affecting immune cell therapy. Among them, cytotoxic T lymphocyte-associated antigen 4 (CTLA-4) and programmed death receptor 1 (PD-1) had the most significant effects. Based on our experience with CAR T-cell therapy, CTLA-4 and/or PD-1 blocking antibodies can greatly improve clinical outcomes if given to patients at the right time after reinfusion of TCR-T cells [318–320]. In addition to these traditional immunosuppressive cells and molecules, some metabolic regulatory molecules also inhibit the function of effector T cells, such as CD73 and indoleamine-2,3-dioxygenase 1 (IDO1) [321, 322]. These immunosuppressive factors in the tumor microenvironment should be considered in future treatment.

#### Tumor heterogeneity

Tumor heterogeneity refers to the genetic and biological differences among different malignant cells within the same tumor tissue [323]. In TCR-T-cell therapy, the influence of heterogeneity is mainly reflected in the difference in antigen expression and presentation. The expression level and immunogenicity of tumor antigens are the basis and key of TCR-T-cell therapy. If the tumor cells do not express or present the target antigen, the TCR-T cells cannot attack those tumor cells [226, 261]. In TCR-T-cell therapy, it is hoped that the target antigen will be highly expressed in most, if not all, tumor cells. However, the reality is that a single target antigen is only expressed in a few malignant cells. This requires treatment with TCR-T cells that have two or more targets [10]. In addition, treatments targeting MHC-Class I and Class II molecularly restricted antigens should also be considered. Multitarget combination therapy may yield more positive results [126]. In addition, tumor-specific antigen expression is often downregulated or even terminated after T-cell infiltration. The alteration of antigen expression is associated with the elimination of specific tumor cells. At the same time, it is also related to changes in key genes in tumor cells, such as the expression and modification of antigen genes, MHC genes and genes related to antigen processing and presentation. Fortunately, some TCR-T-cell target antigens are epigenetically regulated. Demethylation or histone deacetylase drugs can be used to enhance the expression of such antigens and improve the therapeutic effect of T cells [324–327].

#### The number of effector cells

The degree of differentiation, survival time, ability to move to the tumor site and expression level of TCR cells affect the efficacy of cell therapy. In TCR-T-cell therapy, the T-cell type and differentiation level directly affect the function and action time of effector cells. To a large extent, these factors determine the efficacy of TCR-T-cell therapy [176, 260]. First, T cells are activated and eventually differentiate into effector cells (Teff) and memory cells (Tm). On the one hand, in vitro studies have shown that Teff cells are more effective in short-term killing. On the other hand, clinical studies have shown that the survival time of effector cells in the body largely determines the therapeutic effect. This means that Tm cells will achieve better results when used for treatment. This influencing factor is more obvious in CD8^+^ T-cell selection [328–331]. Naive T cells (Tn) can differentiate into stem cell-like memory T cells (Tscm), central memory T cells (Tcm), effect-memory T cells (Tem) and Teff cells when the initial antigen and costimulatory signal reach a certain quality and quantity. Preclinical studies have shown that CD8^+^ T-cell differentiation and proliferation are antithetical. Therefore, it can be inferred that the differentiation degree of CD8^+^ T cells is inversely related to their persistence and therapeutic effect in vivo [332–334]. Currently, two strategies are available to improve T-cell therapy. One involves adding the right cytokines. For example, the addition of IL-7 + IL-15 or IL-15 + IL-21 resulted in the cultivation of poorly differentiated CD8^+^ T cells [335–337]. The other method involves enriching poorly differentiated T cells according to CD62L expression; these cells then act as receptor cells for gene transfer [338, 339]. The proportion of Tm cells in TCR-T cells can be increased, and the survival time of T cells can be prolonged by adjusting the culture scheme and by appropriate gene modification.

In addition, the type of T-cell subsets injected also directly affects the therapeutic effect. The differentiation of naive T cells into functional T cells is influenced by various cytokines in the surrounding environment. In turn, differentiated T cells affect T-cell function. This variability is particularly common in helper T-cell (Th) differentiation. CD4^+^ T cells can differentiate into a variety of subtypes, including Th1, Th2, Th9, Th17, Th22, Tfh and Treg cells [340–342]. Th1 and Th17 are the most functional cell subtypes in terms of antitumor effects. The addition of CD4^+^ T cells, especially Th1 cells, can prevent the exhaustion of CD8^+^ T cells and increase the infiltration of CD8 + T cells, thus leading to effective tumor killing and removal [343, 344]. Th17 cells enhanced the tumor-clearing effect of TCR-T cells in animal experiments, and this effect of Th17 cells was related to their transformation into Th1 cells [345–347]. In the present study, Th cell activity was effectively activated by transferring MHC-I class molecule-restricted TCR and/or CD8 molecules into CD4^+^ T cells. It is possible to enhance the efficacy of cell therapy by controlling the subpopulation of transfused T cells [348, 349]. These results suggest that the combination of CD8^+^ T cells and Th1/Th17 cells can improve the therapeutic effect of T cells.

#### Affinity of T-cell infusion

T-cell affinity refers to the ability of T cells to respond to specific concentrations of antigens [189, 350]. In TCR-T cells, imported TCRs often have high affinity. Therefore, the affinity of TCR-T cells is mainly related to the expression level of TCR. The expression of the TCR transgene was enhanced by optimizing the method of gene transfer, including the selection of the gene transfer method, the use of the best vector components and the use of transgenic boxes [351]. Using lentiviral vectors and EF-1 promoters, it is possible to significantly increase the level and proportion of TCR expression based on the experience of CAR T-cell studies [352]. In addition, TCR gene expression can be enhanced by limiting or eliminating TCR mismatch. Strategies to promote the correct pairing of α and β chains of transgenic TCR (to prevent and reduce TCR mismatch) fall into two main categories. First, the mouse modification of TCR was used as the representative, that is, the C region was replaced with the sequence of mouse origin to prevent the mismatch between human transgenic TCR and its own TCR. Second, the correct pairing is achieved through CRISPR/Cas9-mediated endogenous TCR knockout [353–355]. In addition to enhancing TCR surface expression, TCR affinity can also be enhanced through TCR sequence changes and optimization strategies to improve the therapeutic effect. It was found that the high-affinity MART-1-specific TCR-mediated tumor response rate was significantly better than that of low-affinity TCR [356, 357]. Moreover, NY-ESO-1-specific TCR-mediated therapy with enhanced affinity is more effective [358, 359]. In addition, there are different ways to improve TCR affinity. A typical example is to improve affinity by changing the CDR sequence of TCR. TCR affinity can be improved by changing the types of certain amino acids in the CDR region. Although increasing TCR affinity can significantly improve T-cell function, special attention should be given to potential off-target effects [360, 361]. In clinical trials, enhanced TCR affinity to CEA/HLA-A2, MAGE-A3/HLA-A2, and MAGE-A3/HLA-A1 was found to be associated with toxic reactions in patients [362–364].

#### Expression of chemokine receptors and immunomodulatory receptors in transfused T cells

At present, TCR-T-cell therapy is achieved through intravenous transfusion. T cells must first travel from blood vessels to the tumor site before they can perform their tumor killing function. The key factors influencing T-cell chemotactic movement are directed movement mediated by chemokines and chemokine receptors (ChRs) [365–367]. Therefore, if TCR-T cells can express an appropriate ChR, the infiltration of T cells into tumors can be increased, and the therapeutic effect of TCR-T cells can be improved. However, the regulatory mechanism of ChR expression is not well understood, and it is difficult to induce specific ChR expression by cytokine or agent addition; although, specific ChR can be introduced by means of genetic engineering [368, 369]. A number of studies have confirmed that ChR gene engineering can improve the directed movement of T cells and improve the effect of immunotherapy [370–372]. It is believed that TCR-T-cell therapies will also benefit from such improvements. In addition to ChR receptors, T cells also express a variety of synergistic signaling receptors that play an immunomodulatory role. The representative costimulatory molecules are CD28, ICOS and 4-1BB. These receptors, when combined with corresponding ligands, can provide costimulatory and coinhibitory signals. In the absence of costimulatory signals, T cells are exhausted under constant antigenic stimulation. T cells show decreased proliferative ability and effector function and upregulation of coinhibitory molecule expression. In theory, it should be possible to enhance T-cell function by activating costimulatory molecules. However, clinical studies using the molecule's activated antibodies against tumors have shown the limited effectiveness of such therapeutic strategies. Thus, this may not be the best option for improving the efficacy of T cells in tumor therapy [373–375]. In contrast, the treatment regimen targeting coinhibitory molecules has achieved better results. The representative coinhibitory molecules are CTLA-4 and PD-1 [376, 377]. Clinical studies have shown that blocking PD-1 or CTLA-4/PD-1 at the same time has a synergistic effect with immunotherapy for multiple solid tumors [378, 379]. Therefore, the combination of T cells and coinhibitory molecules should be considered in TCR-T-cell therapy.

### (八)、Overview of clinical trials of TCR-T-cell therapy

Clinical trials of TCR-T-cell therapy began as early as 1998, but an increasing number of clinical trials began after Mart-1-specific TCR-T cells were successfully used to treat melanoma in 2006. Currently, TCR-T cells are being tested in clinical trials for melanoma, synovial sarcoma, rectal cancer, esophageal cancer, and myeloma. More than 100 TCR-T-cell clinical trials have been registered on the Clinical Trials website. Among them, some clinical trials have had research results and published articles reporting their efficacy, which provides an important reference for the future adoptive transfusion therapy of TCR-T cells (Table 3). Additionally, a large number of clinical trials targeting TCR-T-cell therapy are currently recruiting (Table 4).

**Table 3:** List of published TCR-T cell clinical trials

Target antigen	Epitope	HLA type	Cancer type	Number of patients	Number of TCR-T cells	Phase	NCT number	ORR (%)	Clinical response	Toxicities	Author
HPV16-E6	TIHDIILECV	HLA-A*02:01	HPV16-positive epithelial cancer	12	0.1–13.4 × 10^10^	I/II	NCT02280811	2 (17%)	2 PR	None	Doran
HPV16-E7	YMLDLQPET	HLA-A*02:01	HPV16-positive epithelial cancer	12	0.1–12.1 × 10^10^	I	NCT02858310	6 (50%)	6 PR	None	Nagarsheth
MCPyV	KLLEIAPNC	HLA-A*02:01	Merkel cell carcinoma	2	1–9 × 10^8^	I	NCT03747484	1 (20%)	0	None	Veatch
HBV	N.A	HLA-A*02/ HLA-Cw*0801	HBV-HCC	8	1 × 10^4^/kg-5 × 10^6^/kg	I	NCT03899415	1 (12.5%)	1 PR	Liver toxicity; 1 (12.5%)	Meng
NY-ESO-1	SLLMWITQC	HLA-A*02:01	Melanoma (11); Synovial sarcoma (6)	17 (11 + 6)	0.16–13 × 10^10^	I	NCT00670748	5 (45%); 4 (67%)	2 CR; 7 PR	None	Robbins
NY-ESO-1	SLLMWITQC	HLA-A*02:01	Melanoma (20); Synovial sarcoma (18)	38 (20 + 18)	0.9–13 × 10^10^	II	NCT00670748	11 (55%); 11 (61%)	5 CR; 17 PR	None	Robbins
NY-ESO-1	SLLMWITQC	HLA-A*02:01	Melanoma; Synovial sarcoma	10	1 × 10^9^	I	NCT02070406	2 (20%)	2 PR	None	Nowicki
NY-ESO-1	SLLMWITQC	HLA-A*02:01/02:06	Synovial sarcoma	12	0.4–14.4 × 10^9^	I/II	NCT01343043	6 (50%)	1 CR; 5PR	CRS: 5 (42%)	D’Angelo
NY-ESO-1	SLLMWITQC	HLA-A*02:01/02:06	Synovial sarcoma	30	2.67 × 10^9^	I/II	NCT01343043	9 (30%)	9 PR	None	Ramachandran
NY-ESO-1	SLLMWITQC	HLA-A*02:01	Melanoma	3	0.6–7.1 × 10^9^	I	NCT03399448	0	0	None	Stadtmauer
MART-1	EAAGIGILTV	HLA-A*02:01	Melanoma	12	0.05–4.56 × 10^9^	I/IIa	NCT02654821	2 (16.7%)	2 PR	CRS/sepsis: 1 (8%); Dermatitis: 10 (83%); Uveitis: 2 (17%); Hearing loss: 4 (33%)	Rohaan
MART-1	EAAGIGILTV	HLA-A*02:01	Melanoma	13	0.6–4.8 × 10^9^	II	NCT00910650	0	0	Skin rash: 3 (23%); CRS: 2 (15%	Chodon
MART-1	AAGIGILTV	HLA-A*02:01	Melanoma	20	0.15–10 × 10^10^	II	NCT00509288	6 (30%)	6 PR	Skin rash: 14 (70%); Uveitis: 11 (50%); Hearing loss: 10 (50%)	Johnson
MAGE-A3	KVAELVHFL	HLA-A*02:01	Melanoma (7); Synovial sarcoma (1); Esophageal cancer (1)	9 (7 + 1 + 1)	2.8–7.9 × 10^10^	I/II	NCT01273181	5 (56%)	1 CR; 4 PR	Severe neurologic toxicity: 3 (33%) including 2 deaths	Morgan
MAGE-A3	EVDPIGHLY	HLA-A*01	Melanoma (1); Myeloma (1)	2 (1 + 1)	2.4–5.3 × 10^9^	I	NCT01350401; NCT01352286	0	0	Severe cardiac toxicity and death: 2 (100%)	Linette
MAGE-A3		HLA-DPB1*0401	Metastatic solid tumors	17	0.01–123 × 10^9^	I	NCT02111850	4 (23.5%)	1 CR; 3 PR	Liver toxicity: 2 (12%)	Lu
MAGE-A4	NYKRCFPVI	HLA-A*24:02	Esophageal cancer	10	0.2–5 × 10^9^	I	UMIN000002395	0	0	None	Kageyama
MAGE-A4	GVYDGREHTV	HLA-A*02	Advanced solid tumors	38	0.12–10 × 10^9^	I	NCT03132922	9 (23.7%)	9 PR	CRS: 19 (50%)	Hong
MAGE-A10	GLYDGMEHL	HLA-A*02:01/02:06	NSCLC	11	0.1–6.77 × 10^9^	I	NCT02592577	1 (9%)	1 PR	Neurotoxicity: 1 (9%); CRS: 3 (27%)	Blumenschein
gp100	KTWGQYWQV	HLA-A*02:01	Melanoma	16	0.18–11 × 10^10^	II	NCT00509496	3 (16%)	1 CR; 2 PR	Skin rash: 15 (94%); Uveitis: 4 (25%); Hearing loss: 5 (31%)	Johnson
CEA	IMIGVLVGV	HLA-A*02:01	Colorectal cancer	3	2–4 × 10^8^	I	NCT00923806	1 (33%)	1 PR	Severe transient colitis: 3 (100%)	Parkhurst
TP53	HMTEVVRHC	HLA-A*02:01	Metastatic breast cancer	1	5.3 × 10^10^	I	NCT03412877	1 (100%)	1 PR	CRS	Kim
KRAS G12D	GADGVGKSA/GADGVGKSAL	HLA-C*08:02	Metastatic pancreatic cancer	1	1.6 × 10^10^	I	IND27501	1 (100%)	1 PR	None	Leidner
Mutation-associated neoantigens	CRISPR-Cas9 KO-KI	Multiple HLA class I	Metastatic solid tumors	16	0.13–4.0 × 10^10^	I	NCT03970382	0	0	CRS: 1 (6%); Neurotoxicity: 1 (6%)	Foy
WT1	CMTWNQMNL	HLA-A*24:02	AML; MDS	9	0.12–3.5 × 10^9^	I	UMIN000011519	5 (55.6%)	5 PR	None	Isao Tawara

**Table 4:** List of recruiting TCR-T cell clinical trials

Neoantigen	Cancer type	HLA	Cell source	Date	Phase	NCT number	Conditions
HPV16-E6	Cervical Carcinoma	HLA-A2	PB (Patient)	2022/8/10–2021/8/31	I/II	NCT05357027	Recruiting
HPV16-E7	HPV-associated Cancers	HLA-A*02:01	PB (Patient)	2023/3/7–2025/1/1	II	NCT05686226	Recruiting
	HPV-associated Cancers	HLA-A2	PB (Patient)	2017/1/27–2026/1/1	I/II	NCT02858310	Recruiting
HPV16- CRTE7A2-01	Advanced Cervical, Anal, or Head and Neck Cancers	HLA-A2	PB (Patient)	2022/7/17–2024/12	I	NCT05122221	Recruiting
HPV18- HRYZ-T101	HPV18 positive advanced solid tumor	HLA-A*11:01/02:01/24:02	PB (Patient)	2023/3–2025/9	I	NCT05787535	Recruiting
MAGE	Advanced melanoma (MEL) and head-and-neck carcinoma (HNSCC)	HLA-A2	PB (Patient)	2020/10/20–2027/10/20	I/II	NCT04729543 (MAGE-C2)	Recruiting
	Advanced-Stage/Metastatic MAGE-A1^+^ Solid Tumors	HLA-A*02:01	PB (Patient)	2022/7/29–2037/6	I/II	NCT05430555 (MAGE-A1)	Recruiting
	Advanced Solid Tumors	HLA-A2	PB (Patient)	2019/5/17–2025/7	I/II	NCT03973333 (MAGE-A4)	Recruiting
KRAS	Advanced Pancreatic Cancer	HLA-A*11:01	TIL (Patient)	2021/10/21–2025/4	I/II	NCT04146298 (KRAS G12V)	Recruiting
	Advanced Pancreatic Cancer and Other Solid Tumors	HLA-A*11:01	PB (Patient)	2022/6/7–2025/6/30	I	NCT05438667 (KRAS G12V or G12D)	Recruiting
	Pancreatic/Gastric/Gastrointestinal /Colon/Rectal Cancer	HLA-A*11:01	PB (Patient)	2017/9/21–2028/7/29	I/II	NCT03190941 (KRAS G12V)	Recruiting
	Pancreatic/Gastric/Gastrointestinal /Colon/Rectal Cancer	HLA-A*11:01	PB (Patient)	2019/4/16–2028/12/1	I/II	NCT03745326 (KRAS G12D)	Recruiting
NY-ESO-1 (CTAG1A)	Locally Advanced Malignant Neoplasm/ Unresectable Malignant NeoplasmSarcoma	HLA-A*02:01	PB (Patient)	2017/7/26–2024/8/1	I	NCT03240861	Recruiting
	Advanced Soft-tissue Sarcoma	HLA-A*02	PB (Patient)	2022/11/11–2028/12/1	I	NCT05620693	Recruiting
	NY-ESO-1 -Expressing Metastatic Cancers	HLA-A*02	PB (Patient)	2022/41–2027/12/30	I/II	NCT05296564	Recruiting
	Solid Tumors	HLA-A*02:01	PB (Patient)	2022/12–2026/3	I	NCT05648994	Recruiting
	• Soft tissue sarcoma	HLA-A*02:01	PB (Patient)	2022/7/8–2024/9/1	II	NCT05549921	Recruiting
AFP	Advanced hepatocellular carcinoma (HCC) or other AFP expressing tumor Types	HLA-A2	PB (Patient)	2017/4/8–2036/7/7	I	NCT03132792	Recruiting
HBV	HBV related hepatocellular carcinoma	HLA-A*02:01/24:02	PB (Patient)	2022/4/20–2024/7/1	I	NCT04745403	Recruiting
	Hepatocellular carcinoma	HLA-A2	PB (Patient)	2019/4/20–2024/6/1	I	NCT03899415	Recruiting
	Hepatocellular carcinoma; Liver cancer, Adult; Liver cell carcinoma	HLA-A *02	PB (Patient)	2022/6–2027/5	I/II	NCT05195294	Recruiting
	Hepatitis B Virus related hepatocellular carcinoma; Hepatocellular carcinoma recurrent	HLA-A *02	PB (Patient)	2022/10/26–2025/10	I/II	NCT05417932	Recruiting
	Hepatocellular carcinoma	HLA-A*02	PB (Patient)	2021/4/14–2023/12	I	NCT05339321	Recruiting
gp100 (PMEL)	Advanced Melanoma	HLA-A*02:01	PB (Patient)	2022/12/19–2027/9	II/III	NCT05549297	Recruiting
	Malignant Melanoma	HLA-A*02:01	PB (Patient)	2015/11–2025/1	I/II	NCT02535078	Recruiting
HERV-E	Renal cell carcinoma; Kidney cancer	HLA-A*11:01	PB (Patient)	2018/7/20–2032/12/31	I	NCT03354390	Recruiting
EBV	Nasopharyngeal Carcinoma	HLA-A*02:01/24:02/11:01	PB (Patient)	2022/12–2030/10	I	NCT05587543	Recruiting
	Nasopharyngeal Carcinoma	HLA-A*02:01/24:02/11:01	PB (Patient)	2023/3–2023/8	I/II	NCT04509726	Recruiting
	Head and Neck Squamous Cell Carcinoma	HLA-A*02:01/24:02/11:01	PB (Patient)	2019/3/1–2024/1/1	I/II	NCT04139057	Recruiting
	EBV Lymphoma; Post-transplant lymphoproliferative disease (PTLD)	HLA-A*02:01/24:02/11:01	CTLs (Patient)	2023/5–2025/12	I/II	NCT05688241	Recruiting
	Head and Neck Squamous Cell Carcinoma	HLA-A*02:01/24:02/11:01	PB (Patient)	2023/3/1–2027/6/30	I	NCT05697926	Recruiting
PRAME	Select Advanced Solid Tumors	HLA-A2	PB (Patient)	2020/2/25–2026/2	I/II	NCT04262466	Recruiting
HA-1H	Relapsed or persistent hematologic malignancies	HLA-A*02:01	PB (Patient)	2020/7/2–2025/7	I	NCT04464889	Recruiting
LAGE-1a	Neoplasms	HLA-A*02:01/02:05/02	PB (Patient)	2020/12/21–2025/12/4	I	NCT04526509	Recruiting
	Neoplasms	HLA-A*02	PB (Patient)	2019/12/31–2026/7/31	II	NCT03967223	Recruiting
CMV	CMV Infection After Allogenic HSCT	HLA-A*02:01/11:01/24:02	PB (Patient)	2021/10/15–2024/12/31	I	NCT05140187	Recruiting
	Allogeneic hematopoietic stem cell transplantation; CMV infection	HLA-A*11:01/02:01/24:02	PB (Patient)	2021/1/6–2023/8	I	NCT05089838	Recruiting
KK-LC-1	Nonsmall cell lung cancer; Solid tumor, Adult	HLA-A*11	PB (Patient)	2018/12/1–2033/12/30	I	CT03778814	Recruiting
	Gastric/Breast/Cervical/Lung Cancer	HLA-A*01:01 NCT05587543	PB (Patient)	2022/9/26–2028/12/31	I	NCT05483491	Recruiting
	Gastric/Breast/Cervical/Lung and other KK-LC-1 positive epithelial cancers	HLA-A*01	PB (Patient)	2022/3/8–2026/4/6	I	NCT05035407	Recruiting
Neoantigen	Advanced Solid Tumor	HLA-A*11:01/02:01/24:02	PB (Patient)	2019/1/8–2023/12/1	I	NCT03891706	Recruiting
	Malignant Epithelial Neoplasms	HLA-A*11:01/02:01/24:02	PB (Patient)	2022/2/8–2027/2/28	I	NCT04520711	Recruiting
	Malignant Epithelial Neoplasms	HLA-A*11:01/02:01/24:02	PB (Patient)	2023/3/15–2028/3/15	I	NCT05349890	Recruiting
	Solid tumors	HLA-A*11:01/02:01/24:02	PB (Patient)	2022/10/1–2039/6	I	NCT05292859	Recruiting
	Solid tumors	HLA-A*11:01/02:01/24:02	PB (Patient)	2021/9/24–2028/5/28	I	NCT05124743	Recruiting
	Solid tumors	HLA-A*11:01/02:01/24:02	PB (Patient)	2022/4/4–2029/3	I/II	NCT05194735	Recruiting
KSH01	Refractory/Recurrent solid tumors	HLA-A * 02	PB (Patient)	2022/8/19–2027/7/31	I	NCT05539833	Recruiting
	Solid tumors	HLA-A * 02	PB (Patient)	2022/12/11–2027/12/11	I	NCT05580796	Recruiting
KSX01	Solid tumors	HLA-A * 02	PB (Patient)	2023/3/7–2028/12/31	I	NCT05811975	Recruiting
Mesothelin	Etastatic pancreatic ductal adenocarcinoma	HLA-A*02:01	PB (Patient)	2021/12/14–2024/6/1	I	NCT04809766	Recruiting
MCPyV	Metastatic or unresectable merkel cell cancer	HLA-A*02	PB (Patient)	2019/7/3–2025/12/15	I/II	NCT03747484	Recruiting

#### TCR-T cells for hematological tumors

Although few clinical studies have been carried out, TCR-T-cell therapy has achieved good results in hematological tumors. The engineered high-avidity TCRs render CD8^+^ T cells specifically cytotoxic to neoantigen-containing tumors. A 2012 trial (NCT01640301) attempted to treat acute myeloid leukemia with WT1 high-affinity CD8^+^ T cells, but the trial is currently closed for enrollment, and the results have not been reported. In 2018, another clinical study (NCT02770820) used WT1-specific central memory and infantile CD8^+^ TCR-T cells to treat acute myeloid leukemia. At the same time, another clinical trial (NCT03326921) using HA1-specific memory TCR-T cells for the treatment of relapsed acute mixed leukemia enrolled patients. The TCRs specifically targeting recurrent fusion genes CBFB-MYH11 confer CD8^+^ T cells antileukemic activity in vitro and in patient-derived murine xenograft (PDX) models with fusion gene-driven AML [46]. Similarly, peripheral blood lymphocytes transduced with TCRs highly reactive to the mutated KRAS variants G12V and G12D could recognize multiple HLA-A*11:01^+^ pancreatic cell lines bearing the appropriate KRAS mutations in a xenograft model (IND27501) [380]. In 2015, the University of Maryland School of Medicine, the University of Pennsylvania School of Medicine and the Adaptimmune Corporation jointly conducted the study of NY-ESO-1-specific TCR-T-cell therapy. Of the patients with multiple myeloma, 80% had a good clinical response, 70% achieved complete or nearly complete response, and the mean progression-free survival reached 19 months [358].

#### TCR-T cells for melanoma treatment

Melanoma is one of the most malignant tumors in skin cancer. Once metastasis occurs, it is insensitive to postoperative adjuvant radiotherapy and chemotherapy, and the prognosis is very poor. At present, TCR-T cell immunotherapy has the most clinical studies in melanoma, and the target is MART-1, P-gp100, NY-ESO-1, MART-A3, and p53 [381]. The large-scale trial of TCR-T cells began in 2006 with two trials targeting metastatic melanoma. Among them, Duval et al. treated 15 patients with melanoma differentiation antigen MART-1-specific TCR-T cells. Although only one patient experienced a partial response, the report demonstrates that TCR-T-cell therapy is safe in humans. Subsequently, Morgan et al. reported the clinical study results of MART-1-specific TCR-T cells used in the treatment of 17 melanoma patients and found that TCR-T cells amplified in vitro could survive in patients for a long time (up to 2 months at the time of detection) and even lasted at a high level in 2 patients for as long as 1 year. Objective responses were achieved in these two patients. Since then, with the deepening of TCR-T-cell research, researchers have realized that TCR affinity is closely related to the therapeutic effect. When high-affinity TCR-T cells were used, patient response rates increased substantially. Johnson et al. used MART-1 high-affinity TCR-T cells to treat 20 patients with metastatic melanoma and found that the objective response rate after treatment reached 30% (NCT02654821; NCT00910650; NCT00509288). Another important factor affecting the efficacy of TCR-T cells is the expression of specific antigens in the tumor. The more widely the antigen is expressed, the better the therapeutic effect of specific TCR-T cells. A typical example is the CT antigen NY-ESO-1. The expression of NY-ESO-1 is very common in tumors. Robbins et al. observed the efficacy of NY-ESO-1-specific TCR-T cells in the treatment of melanoma, and the results showed that most melanoma patients had an objective clinical response, and the 3-year and 5-year survival rates of patients with melanoma were 33%. Since TCR-T cells require costimulatory signals to activate and proliferate, DC cells can enhance the therapeutic effect of TCR-T cells. Chodon et al. observed the effect of a DC vaccine combined with Mart-1-specific TCR-T cells on 13 patients with metastatic melanoma, and the results showed that up to 69% of patients were significantly better (NCT00670748; NCT00670748; NCT02070406; NCT03399448).

In addition, Morgan et al. transferred the TCR gene with MAGE-A3 as the target antigen into T cells for the treatment of metastatic melanoma patients. The results of phase I/II clinical trials showed that the disease response rate was 57% (4/7), of which 1 patient received CR and lasted for 15 months, and 3 patients received PR (of which 2 patients had PR lasting for 4 months and 1 patient had PR lasting for more than 12 months). However, three patients in this clinical trial developed mental disorders caused by brain damage, two of whom developed severe central nervous system damage and died from multifocal necrotizing leukoencephalopathy, which may be caused by neurotoxicity mediated by cross-recognition of the MAGE-A12 antigen in normal brain tissue (NCT01273181;NCT01350401; NCT01352286; NCT02111850). Johnson et al. used melanoma-associated antigen peptides P-gp100 and p53 to prepare TCR-T cells with high expression of TCR-activity for the treatment of metastatic melanoma patients, and observed disease response rates of 18.75% (3/16) and 0% (0/10), respectively. The former had PR in 2 patients and CR in 1 patient, and no adverse reactions were mentioned (NCT00509496; NCT03412877).

#### TCR-T cells for digestive system tumor treatment

Rosenberg's team used mutant antigen-specific CD4^+^ T cells and CD8^+^ T cells to treat patients with cholangiocarcinoma and colon cancer, respectively, with good results. In 2017, the research team conducted clinical trials of HLA-DPB1-restricted MAGE-A3 antigen-specific TCR-T cells. The study yielded promising results, with one patient with esophageal cancer achieving partial remission for up to four months (NCT01273181). However, there are still significant adverse reactions in clinical trials of TCR-T cells. For example, in a 2011 CEA antigen-specific TCR-T-cell clinical trial, three patients developed different degrees of colitis, mainly due to the ability of these high-affinity antigen-specific TCR-T cells to recognize normal colon epithelial tissue expressing CEA, in what is known as an off-target phenomenon (NCT00923806).

AFP is the most widely studied target antigen in HCC. AFP is a glycoprotein synthesized in the liver during the fetal period, and its level in adult serum is very low, but the liver cells with malignant changes can recover its synthesis ability. Therefore, AFP can be used as a target of cellular immunotherapy as a specific antigen of liver cancer cells. HLA-A2^+^/AFP specific TCR has been obtained, which can recognize AFP158-166 peptide, and X-scan detection shows that it does not cross-react with other peptides in the human protein database to avoid off-target effects. Clinical trials of TCR-T for HCC are currently underway to confirm its efficacy by examining AFP expression and T cell infiltration in biopsy tissues and closely monitoring biochemical markers in the liver (NCT03132792; NCT02686372; NCT02719782; NCT04677088). The existing NY-ESO-1-specific TCR for the recognition of NY-ESO-1157 is most prominent, and it has been reported that the TCR-T of NY-ESO-1157 can produce a response in HCC patients, especially in patients with Treg cell depletion. In addition, NY-ESO-1 is highly expressed in tumors of some HCC patients. Therefore, some are also used in clinical treatment trials of HCC (NCT01967823; NCT02869217; NCT03159585). Hepatitis B virus (HBV) and hepatitis C virus (HCV) infection is one of the main causes of HCC, and the expression of virus-related proteins in tumor tissues of patients can be used as target antigens for TCR-T therapy. In 2011, researchers identified HBsAg specific TCR and conducted several clinical treatment trials. Because HBsAg is also expressed in non-HCC tissues, its tumor specificity is low, so the above trials are limited to prevention of recurrence in HCC patients after liver transplantation (NCT03899415).

#### TCR-T cells for the treatment of synovial cell sarcoma

Synovial cell sarcoma is an extremely rare malignant tumor in soft tissue cancer. At present, it is mainly treated by surgery. It is characterized by high local invasiveness and metastasis rate, and the prognosis of patients with metastasis is poor. ROBBINS et al. transferred the TCR gene specific to NY-ESO-1 cancer testis antigen into autologous T cells for the treatment of patients with synovial cell sarcoma. Phase I clinical trial results showed that the disease PR rate was 66.7% (4/6), of which 1 patient's PR duration lasted for 18 months, and no patients who underwent this clinical trial had significant adverse reactions. Four years later, Robbins et al. again used TCR-T cells targeting NY-ESO-1 to treat patients with synovial cell sarcoma, and the results of phase II clinical trial showed that the response rate was 61% (11/18). Among them, 10 patients achieved PR and 1 patient achieved CR more than 20 months; The 3-year and 5-year survival rates were 38% and 14%, respectively, and all patients had transient neutropenia and thrombocytopenia, but no significant adverse effects were found (NCT01343043; NCT03697824; NCT03967223). In addition, Morgan et al. transferred the TCR gene with MAGE-A3 as the target antigen into T cells for the treatment of a patient with synovial cell sarcoma. The results showed that the PR duration of the disease lasted for 5 months without any symptoms of neurotoxicity. TCR-T cell immunotherapy can prolong the survival of patients with synovial cell sarcoma, but there are still some patients who are not responsive to treatment, and the mechanism has not been clarified. Especially for the NYESO-1 target antigen expressed by most malignant tumors, there are still some patients who do not show therapeutic responsiveness when receiving TCR-T cell immunotherapy. This suggests that there may be other factors (such as tumor antigen deletion and low expression, etc.) worthy of consideration and exploration, and finally find an appropriate way to avoid immune escape of TCR-T cells (NCT02869217; NCT03250325).

#### TCR-T cells for the treatment of other types of tumors

In addition to the treatment of malignant melanoma patients and digestive system tumors, TCR-T cells have achieved good clinical efficacy in the treatment of some other tumors, such as esophageal cancer, multiple myeloma, metastatic cervical cancer, metastatic colorectal cancer, urothelial cancer, osteosarcoma, and breast cancer, and still achieved good clinical efficacy. In the TCR-T clinical study on esophageal cancer, Kageyama et al. transferred the TCR gene with MAGE-A4 as the target antigen into T cells for the treatment of 10 patients with esophageal cancer, among which 3 patients survived for more than 27 months. Seven patients developed disease progression (PD) within 2 months of treatment, but no treatment-related adverse effects were observed. However, similar studies conducted by Davis et al., and Morgan et al., did not find a significant therapeutic effect, and patients in the latter study died after coma. The safety and efficacy of autologous T cells that have been engineered to express TCRs particularly targeting the HLA-A*11:01-presented public neoantigens, KRAS-G12V or G12D, are investigated in a clinical trial enrolling patients with advanced pancreatic cancer (NCT04146298, NCT05438667). Moreover, autologous T cells engineered with personalized neoantigen-specific TCRs are also being conducted in solid tumors, such as ovarian cancer, lung cancer, colorectal cancer, pancreatic cancer, cholangiocarcinoma and gynecologic cancer (NCT05292859, NCT05194735, NCT04520711).

### 七、Alternative treatment strategies based on neoantigens

**Fig. 4:**
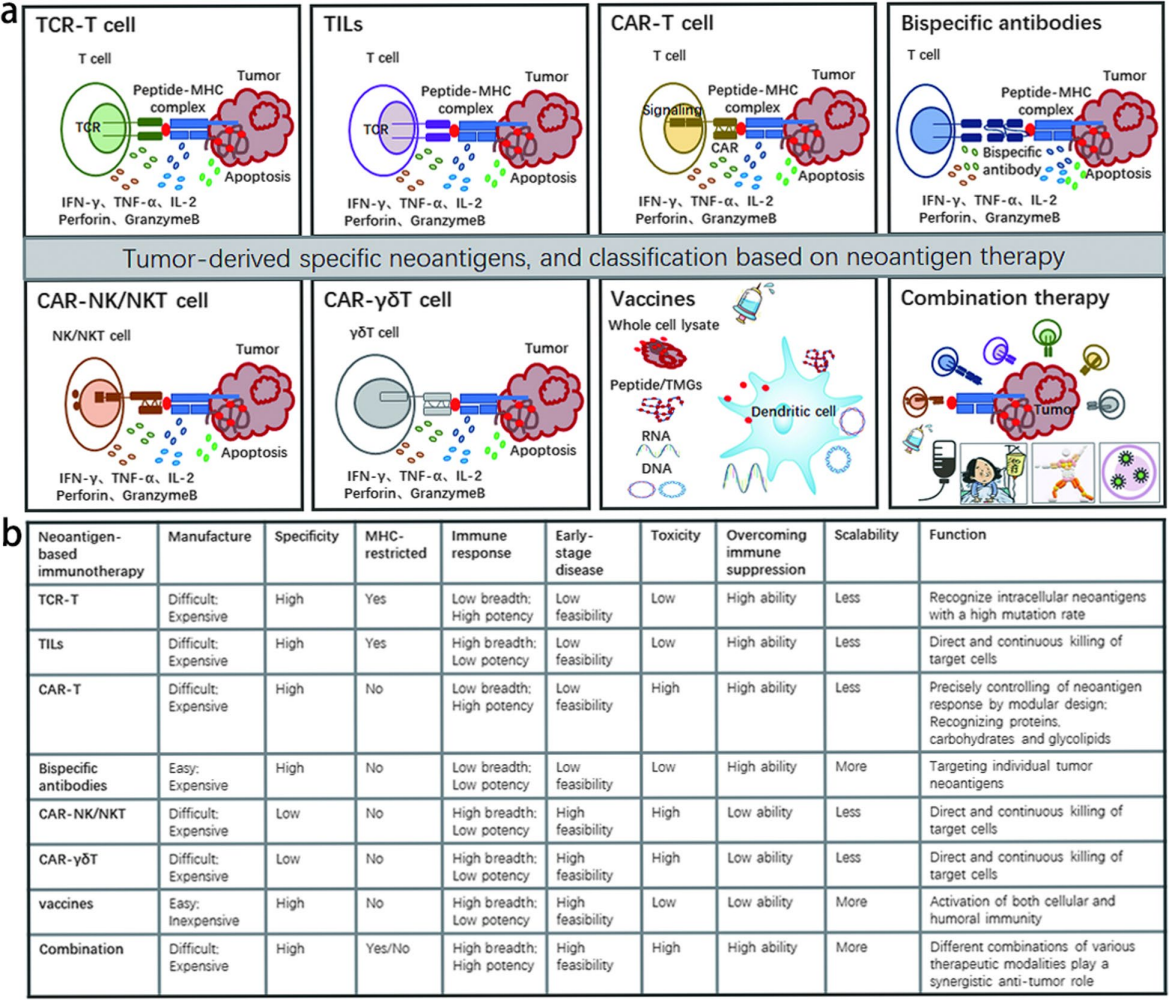
Classification and characteristics of neoantigen-based therapy. **a** Immunotherapies targeting neoantigens mainly include ACTs (TCR-T, TILs, CAR-T, CAR-NK/NKT, CAR-γδT), bispecific antibodies, cancer vaccines and combination therapy regimens. **b** Advantages and disadvantages of neoantigen-based immunotherapy

Based on the advantages of tumor specificity and immunogenetics, neoantigens can become new targets for cancer immunotherapy, including tumor vaccines, ACT and antibody-based therapies, as well as potential predictors of ICB responses. Target neoantigens include individual neoantigens for each patient or common neoantigens expressed in many patients' cancers. Off-the-shelf therapies based on public neoantigens consume less resources and time than individualized neoantigen therapies. Because personalized neoantigens are patient specific, they cannot be used for large numbers of patients. With recent advances in high-throughput sequencing, personalized neoantigens enable the immune system to target the appropriate immunogenic epitope of a malignant tumor without a predefined public antigen (Fig. 4).

### (一)、Therapeutic vaccines based on neoantigens

Neoantigen vaccines have the advantages of high feasibility, high safety and simple production processes. They are an effective way to stimulate, enhance and diversify the antitumor T-cell immune response [382, 383]. Different forms of neoantigen-based vaccines (such as peptides, nucleic acids, and DC vaccines) are being evaluated in clinical trials in patients with different tumor types. Current peptide and nucleic acid vaccines target neoantigens derived from somatic mutations, including SNVs, frameshift INDELs, and gene fusion. DC vaccines can target selected neoantigens either through the synthesis of peptides or nucleic acid pulses or can target the entire TSA through the introduction of whole cell lysates [384–389]. Therefore, neoantigen vaccines are becoming a new type of tumor immunotherapy.

### (二)、Adoptive therapy of TILs

CD8^+^ T lymphocytes have the ability to recognize and eliminate cancer cells, and these lymphocytes were discovered more than 50 years ago [390]. Adoptive transfer of auto TILs amplified in vitro without genetic modification has been shown to induce complete remission in some human cancers [257]. These TILs are extracted from patients, amplified under specific circumstances, and prepared to enhance their anticancer activity [391]. The cell product is then injected back into the same patient. These patients had previously received nonmyeloablative lymphoid clearance chemotherapy and subsequent cytokine therapy, such as with IL-2, which stimulated a strong antitumor immune response [392, 393]. TILs have a rich specificity for neoantigens and are superior to unselected TILs in achieving complete and lasting tumor regression. Most neoantigen-specific TCRs exhibit significantly higher affinity, even for homologous antigens expressed at relatively low levels, compared to the low affinity of tumor antigen-specific TCRs [350, 354]. Therefore, TILs have been used to treat patients with metastatic malignancies who do not respond to current therapies, including chemotherapy, radiotherapy, and anti-PD-1 therapy.

### (三)、CAR-T therapy

CAR T-cell approaches have other advantages over TCR-T cells. They do not depend on HLA expression and neoantigen presentation, which are mechanisms commonly exploited by cancer cells for immune escape [394, 395]. The engineered expression of CAR molecules includes an intracellular signaling and co-signaling domain, as well as an extracellular antigen-binding domain, that enables CAR T cells to bind to any cell surface protein with antibodies and then activate MHC-independent CAR T cells. Boolean logic gates can be used in CAR T cells even if the antigen is not completely specific. These gates improve the specificity of tumor recognition by activating tumor-specific neoantigens and improves the clearance efficiency of tumor cells by targeting tumor-expressed antigens [396–398]. Early clinical trials using CD19-targeted CAR-T cells for the treatment of B-cell malignancies patients showed outstanding results, while CAR-T cells for the treatment of patients with solid cancer showed poor outcome because of the limited antigens. Tumor neoantigens have inspired creative solutions and given solid tumor patients hope for CAR-T therapy. The limited number of tumor-specific surface neoantigens that are suited for CAR-T can be overcome by integrating a single-chain variable fragment (scFv) that recognizes a neoantigenic pMHC complex on the tumor surface. CAR-T cells redirected at novel neoantigens are being tested in ongoing clinical trials in hematological and solid tumors [399, 400]. A CAR that can recognize the EGFRvIII neoantigen has been created as a part of a lentiviral vector and a truncated EGFR that lacks the ligand binding domain and cytoplasmic kinase domain is incorporated for in vivo tracking and ablation of CAR-T cells in necessary. Human EGFRvIII^+^ xenogeneic subcutaneous and orthotopic models showed that EGFRvIII-directed CAR-T cells could control tumor growth [401]. The T cells can generate CARs that target antigens universally expressed by tumors, like EphA2 and IL13R2, after being primed by a highly tumor-specific neoantigen, like EGFRvIII, and being trained to carry out complete tumor destruction [402]. CAR-NK/NKT and CAR-γδT cells are also being developed as therapies [403].

### (四)、Antibody therapy against neoantigens

In contrast to conventional antibodies that cannot target intracellular proteins, TCR-mimic (TCRm)- or mutation-associated neoantigen (MANA)-specific antibodies can recognize intracellular neoantigens by focusing on the pMHC complex. TCRm antibodies have a stronger binding affinity than TCR, which has been shown to be the key to minimizing on-target, off-tumor effects [404–407]. These antibodies targeting neoantigens are readily converted into a variety of therapeutic forms, including full-length antibodies, antibody‒drug conjugates (ADCs), and bispecific antibodies (BsAbs) [408–411]. Phage display, yeast display, and genetic platforms are some of the techniques used to detect human TCRm antibodies that have extremely high specificity for neoantigens presented on HLA. For example, BsAbs can be used to solve the problem of the density of the mutant p53/pMHC complex on the cell surface being insufficient to recruit T lymphocytes to the tumor site [412]. The bispecific T-cell engager (BiTE) is a BsAb constructor that provides an effective and powerful signal for T-cell activation by simultaneously binding neoantigens on tumor cells and CD3 complexes on T cells [413, 414]. Thus, strategies based on TCRm antibodies can be used to target neoantigens derived from oncogene and TSG mutations that are difficult to eradicate using conventional methods. This could lead to more targeted anticancer therapies. The affinity of TCR-Mimic antibodies to polypeptide-HLA molecules is much better than that of natural TCR. To prevent cross-reactivity or the binding of HLA components unrelated to a given peptide, TCR-mimic antibodies must be screened appropriately. Similar to designed TCRs, cross-reactivity can be prevented by negative selection of off-target peptides. At least one of the synthetic agents showed lower cross-reactivity than the equivalent natural receptors [415–417].

### (五)、Neoantigen-based combination therapy

**Fig. 5:**
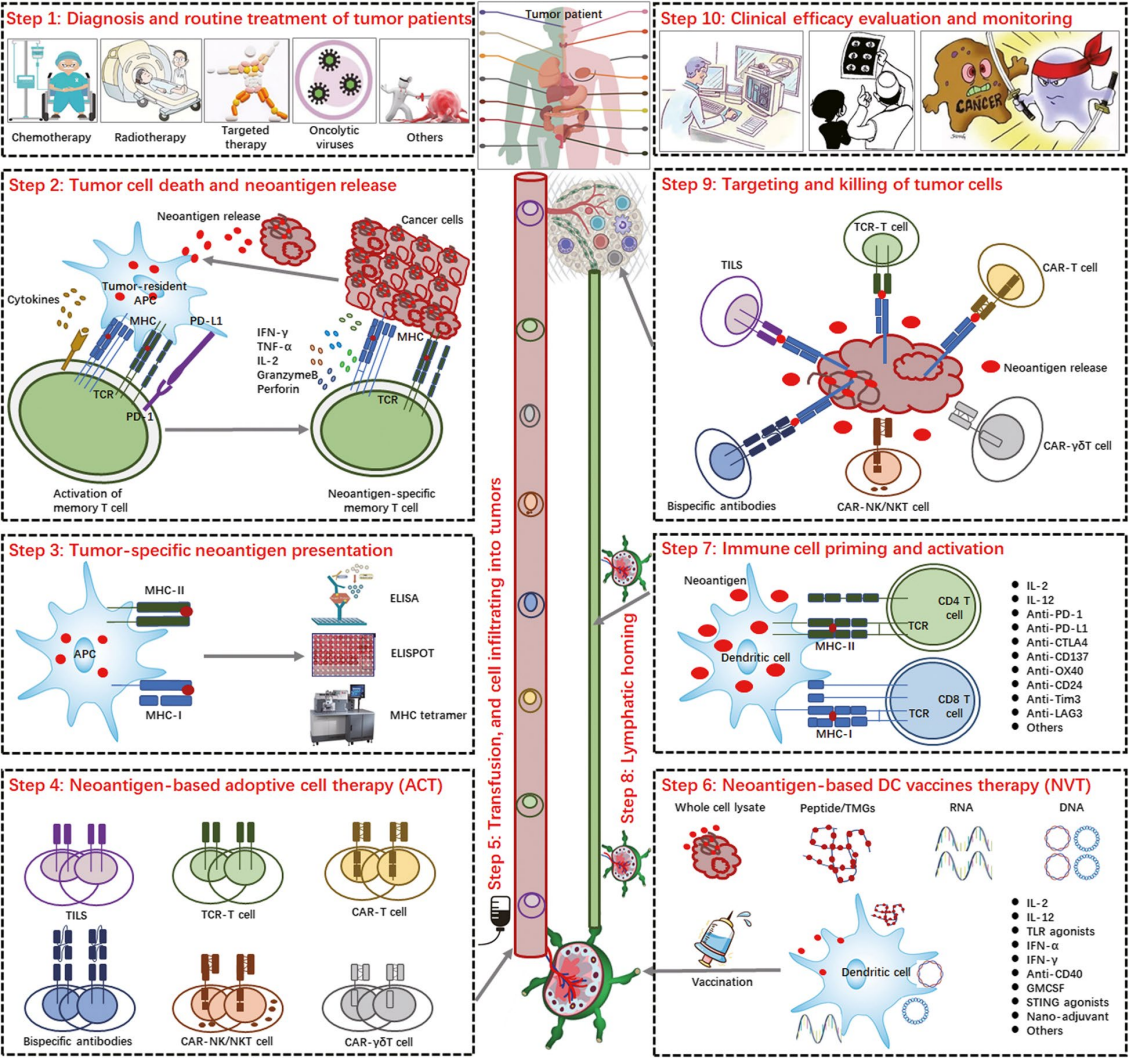
Combined antitumor strategies based on neoantigens. The diagnosis and routine treatment of tumor patients (Step 1). The formation of tumor cells initiates the immune function of T cells, and the tumor cells die and lyse, resulting in the release of neoantigens (Step 2). Neoantigens produced by tumors are released and captured by DCs. The DC transmits the collected neoantigens on the MHC-I and MHC-II molecules to the T cells (Step 3). Immunotherapies targeting neoantigens (neoantigen-based adoptive cell therapy) mainly include TCR-T cells, TILs, CAR-T cells, CAR-NK/NKT cells, CAR-γδ T cells and bispecific antibodies (Step 4). Adoptive back transport of ACT cells and chemotaxis into the tumor play an antitumor role (Step 5). Neoantigen-based DC vaccine therapy is also initiated (Step 6). Immune cells are primed and activated in the lymph node (Step 7). Effector cells develop into effector memory cells through lymphatic homing (Step 8). Effector memory ACT cells target and kill tumor cells (Step 9). After a series of treatments, clinical evaluation and efficacy monitoring are performed (Step 10). In brief, the “Cancer-Immunity Cycle” includes enhancing neoantigen release by chemotherapy, radiation therapy and oncolytic viruses, increasing the quantity and quality of tumor-reactive T cells through cancer vaccines and ACTs, and boosting the infiltration and cytotoxicity efficacy of immune cells via checkpoint inhibitors

Due to the heterogeneity of neoantigen profiles and the evolving immune escape mechanisms of cancer cells, immunotherapy alone is not effective in the treatment of advanced cancer patients [269, 418]. The combination of several immunotherapies can simultaneously target different stages of the cancer immune cycle, including antigen release and presentation, immune cell initiation and activation, immune cell metastasis and invasion of the tumor, and the recognition and killing of cancer cells, thereby enhancing anticancer efficacy. For example, neoantigen-based immunotherapy combined with ICBs, neoantigen vaccines combined with ACT therapy, neoantigen-based immunotherapy combined with traditional therapy, etc. Another strategy is to combine therapies with different mechanisms to overcome resistance induced by tumor heterogeneity [269, 292, 407, 419–424]. All targeted cancer cells must have the same pattern of neoantigen expression and presentation; otherwise, resistant clones with no predicted neoantigens can survive and confer clonal growth advantages. Thus, precision immunotherapy can be combined with conventional treatments, such as chemoradiotherapy, to kill cancer cells without relying on neoantigens. This can lead to more significant and lasting therapeutic effects [425, 426]. The "cancer-immune cycle" refers to the sequence of events that must be initiated, conducted, and expanded to achieve an anticancer immune response that effectively eradicates cancer cells. In short, neoantigens produced by tumor formation are released and captured by DCs. The DC transmits the collected neoantigens on the MHC-I and MHC-II molecules to T cells, thereby initiating and activating effector T-cell responses against cancer-specific neoantigens. The activated effector T cells then migrate and infiltrate the tumor bed, where they recognize and ultimately destroy cancer cells. The death of cancer cells produces more tumor-associated neoantigens that amplify and enhance the immune response in subsequent cycles [427–430]. Thus, cancer immunotherapy aims to restart or amplify the self-sustaining cancer immune cycle. A variety of immunotherapies have been developed to target rate-limiting steps in the tumor immune cycle, including enhancing neoantigen release through chemoradiotherapy and oncolytic viruses, increasing the number and quality of tumor-reactive T cells through cancer vaccines and ACTs, and enhancing immune cell invasion and cytotoxic effects through checkpoint inhibitors [108, 431–435]. Therefore, precision immunotherapy can be combined with conventional treatments like radiotherapy and chemotherapy that kills cancer cells independent of the neoantigens, achieving a more prominent and durable therapeutic effect (Fig. 5).

#### Neoantigen-based immunotherapies and ICBs

Checkpoint inhibitor-based immunotherapy has achieved prolonged anti-tumor effects in several malignancies, including renal cell carcinoma, NSCLC and melanoma. Patients, however, do not react to ICB therapy in the absence of tumor-specific effector T cells. Moreover, ICB therapy only affects one or two phases of the anti-cancer immunity pathways, such as anti-CTLA4 antibodies regulate the immune cell priming and activation, while anti-PD-1/PD-L1 antibodies focus on the final negative regulation of T effector cells [436, 437]. ICBs enhance specific T cell responses by targeting neoantigens, including PRKDC, EVI2B and S100A9, in a relapsed multiple myeloma patient. For patients with solid tumors who are unresponsive to, or relapsed following anti-PD-1 therapy, mRNA-based neoantigen vaccines, such as mRNA-4157, mRNA-5671, and BNT122, are used together with immune checkpoint inhibitors in multiple clinical trials. The anti-tumor efficacy of CTLs, including those specific for mutation-associated neoantigens, can be further boosted by ICB therapy [438]. In addition, Persistent exposure to TSAs promotes the exhaustion of CD8^+^ T cells, which characteristically expressed high levels of PD-1 and CD39. ICBs can reinvigorate the exhausted neoantigen-specific T cells via overcoming the suppressive microenvironment [439].

#### Combinations of neoantigen vaccine and ACT

Combinations of neoantigen vaccination and ACT have also been utilized successfully to boost clinical efficacy in tumor treatment. Vaccination can increase the amount of neoantigen-reactive T cells in circulation, possibly by boosting better outgrowth of T lymphocytes. Alternatively, the vaccines can induce de novo T cell responses that overcome the insufficient recognition of neoepitope by T cells due to inadequate cross-presentation of a neoantigen by tumor cells. Vaccine is also used to enhance the efficacy of CAR-T therapy to eliminate solid tumors [440, 441].

#### Neoantigen-based immunotherapies and conventional therapies

The majority of chemotherapeutic agents and radiation therapy were designed based on their direct cytotoxic effects without considering their impact on immune system. Chemotherapy and radiotherapy can be used to increase the release of tumor-specific neoantigens, circumventing issues such as an insufficient number of neoantigens to stimulate T cell response. During the chemotherapy and targeted therapy, the tumor cells often occur new mutations, including reversion mutation, contributing to drug resistance. Many reversions are predicted to encode tumor-specific neoantigens, offering a potential strategy for combating resistance with CAR-T cell therapies, immune checkpoint inhibitors or anti-cancer vaccines [270, 442, 443].

### 八、Limitations of TCR-T-cell therapy

**Fig. 6:**
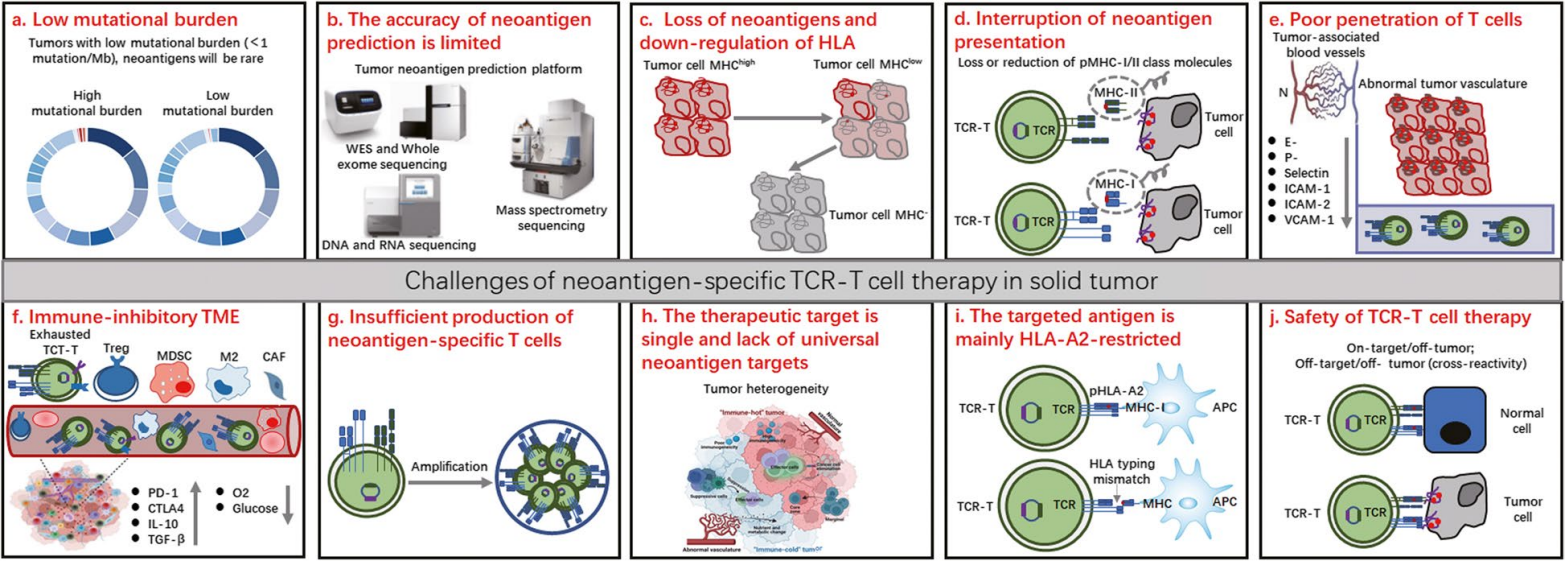
Challenges in the clinical application of neoantigen TCR-T-cell therapy. **a** Low neoantigen load results in a lack of suitable neoantigen targets. **b** At present, the accuracy of neoantigen prediction technology is limited. **c** Downregulation of MHC expression causes tumor cells to lose neoantigen targets. **d** The loss of pMHC molecules leads to the interruption and reduction of neoantigen presentation. **e** The expression of adhesion molecules and stroma-rich and abnormal blood vessels in tumor tissues is downregulated, which limits the effective penetration of T cells. **f** Immunosuppressive tumor microenvironments inhibit T-cell function. **g** The technical bottleneck of ACT leads to the production of neoantigen-specific T cells. **h** Tumor heterogeneity leads to the singleness of specific tumor therapeutic targets and the absence of universal neoantigen targets. **i** The neoantigen epitopes developed thus far are mainly for HLA-A2 targets. **j** Safety of TCR-T-cell therapy itself

Despite success in hematological malignancies and solid tumors, neoantigen-based immunotherapies have shown objective efficacy in only a few documented patient responses. Therefore, a considerable number of changes are needed to improve clinical outcomes, including increasing the accuracy of neoantigen prediction, overcoming immune evasion, and optimizing the production process (Fig. 6).

### (一)、The accuracy of neoantigen prediction is limited

The widespread use of personalized immunotherapy is limited by limitations in the discovery of neoantigens that target tumors. This is due to the heterogeneity of mutation load and the significant differences in neoantigen presentation between different tumor types [444]. Therefore, the identification and prediction of neoantigens should be carried out for specific individuals with cancer. The prediction of neoantigens is also limited by genetic heterogeneity, especially different somatic mutations within different cancer types, different individuals and even different tumor subclones. Only 10% of nonsynonymous tumor cell mutations produce mutant peptides with high MHC affinity, and only 1% of MHC-binding peptides are recognized by patient T cells [445–447]. In addition, the heterogeneity of mutations within tumors provides additional complexity to neoantigen prediction. The genome of tumor cells has undergone extensive generation, cloning, alteration and mutation loss. At the same time, the diversity of neoantigen-specific T cells present in patients may not be fully captured by a single resected lesion due to the limited infiltration of T lymphocytes. This limits the library of TCRs that can be built for therapeutic purposes [448, 449]. Theoretically, the higher the TMB, the more neoantigen-specific T cells are detected in the tumor, resulting in a higher immunotherapy response rate. However, low TMB in hematologic malignancies and some epithelial cancers can also produce neoantigen-responsive lymphocytes. Inadequate neoantigen density in low-TMB malignancies requires more robust strategies to accurately identify novel immunogenic epitopes that CD8^+^ T cells can detect [450–453]. Therefore, there may be tumor clones that do not respond to neoantigen-specific T cells. Because of the selection advantage, these cloned cells may outperform other cloned cells, limiting the clinical benefit.

### (二)、Evasion of immune surveillance

Tumors can evade neoantigen-based immunotherapy through many mechanisms, including neoantigen loss, modification of antigenic peptide presentation, and the immunosuppressive tumor immune microenvironment (TME).

#### Neoantigen loss

Loss of tumor-specific neoantigens may be an important strategy for tumor immune escape. In particular, many neoantigens are byproducts of tumorigenesis and do not play a critical role in tumor cell survival [454]. Neoantigen depletion may also be an intractable mechanism of antitumor immunity, which limits the application of personalized neoantigen-specific immunotherapy. Neoantigen depletion can have a variety of causes, such as copy number loss, transcriptional inhibition, epigenetic silencing, and posttranslational mechanisms. Neoantigens present only in specific tumor cell subpopulations may also be lost due to eradication of entire subclonal cell populations mediated by CD8^+^ T cells. Many of the missing mutations are recognized by the patient's T cells, and neoantigen coding genes are unlikely to be produced in tumors with widespread immune cell penetration. This suggests that neoantigen-expressing tumor subclones may be preferentially removed by the immune system [455, 456]. In addition, loss of neoantigens due to deletion of chromosomal regions or elimination of tumor subclones may lead to acquired resistance to immunotherapies such as ICBs [457, 458]. 因Therefore, to compensate for the loss of targeted neoantigens in immunotherapy, personalized neoantigen-specific immunotherapy should target multiple neoantigens, thereby expanding the range of neoantigen response [292, 459].

#### Interruption of neoantigen presentation

Tumors may undergo mutations that alter not only neoantigen expression but also HLA heterozygosity and MHC stability in response to tumor immune pressure. These changes impede the processing and presentation of neoantigens, which inhibit T-cell recognition and tumor killing. If the key antigen presenting gene beta-2 microglobulin (β2m) is mutated or lacks HLA allelic heterozygosity, the tumor may be able to avoid recognition by adoptive metastatic T lymphocytes [460–462]. The second demonstrated mechanism of epitope loss is the downregulation of MHC molecule expression in tumor cells due to abnormal transcription, translation, or protein stability events [463]. Together, these mechanisms may help partly explain why higher neoantigen loads in some cancers are not associated with better prognosis due to reduced neoantigen presentation. Based on these findings, ICBs may be more effective in cancer treatment if MHC-I presentation is activated using splicing inhibitors or autophagy inhibitors [464–466].

#### Immunosuppressive TME

Tumor cells live in a heterogeneous microenvironment composed of invasive and resident host cells, secretory factors, and extracellular matrix. Infiltrating cells in the TME include T cells (TILs and Tregs), B cells, fibroblasts, macrophages (M1 and M2), MDSCs and other immune cells and secretion factors, including the immunosuppressive cytokines IL-10 and TGFβ. These components can interact with each other to induce a supportive environment for malignant cell growth, migration, and metastasis, thereby escaping the immune system and tumor-specific CTLs [467–469]. Immunosuppressive TME processes can also impair neoantigen recognition and T-cell activation, including suppression of immune checkpoints, immunosuppressive effects of various TME cells, and release of ions or proteins within tumor cells. Immunosuppressive checkpoint ligand molecules (such as PD-L1 and CTLA-4) can biologically limit T-cell growth and function and are commonly increased in tumor cells during immunotherapy [470–473]. Induced neoantigen expression and combined CAR and epitope diffusion are compensation strategies that can be used to address the immune escape of tumor cells. MHC-I immune epitopes can expand by splicing new epitopes due to defective interaction of the splicing complex with RNA, incorrect degradation of auxiliary splicing factors, or abnormal splicing factor PTM [273, 474, 475]. Most tumor stromal cells in the TME express the immunosuppressive checkpoint ligand PD-L1, which can interact with PD-1 expressed on T cells, leading to antitumor functional inhibition and depletion of adoptive metastatic TILs, CAR T cells and TCR-T cells. This effect can be mitigated by blocking checkpoints with anti-PD-1 and anti-PD-L1 antibodies [377, 470]. CTLA-4 expressed on activated T cells has a similar effect because CTLA-4 binds to CD80/86 on antigen-presenting cells with a higher affinity, competing with the T-cell costimulatory molecule CD28 to suppress antitumor immunity. Anti-CTLA4 antibodies not only block the interaction between CTLA4 and CD80/86 but also consume Tregs, thereby promoting the costimulation and amplification of tumor-specific CTLs and improving clinical benefits [376, 476].

Among the multiple immunosuppressive factors secreted by the TME, TGF-β plays a central role in driving tumor signaling, remodeling, and metabolism. TGF-β is produced by many cell types, including tumor cells, stromal cells, and Tregs. It stimulates autocrine and paracrine signaling to promote angiogenesis, inhibits the antitumor response of CD8^+^ and Th1 cells, and induces the epithelial-mesenchymal transformation of tumor cells, thus promoting tumor invasion. Thus, blocking TGF-β signaling in the TME enhances the antitumor response [477, 478]. Indeed, one possible mechanism for anti-CTLA4 antibody therapy is the depletion of immunosuppressive TGF-β-producing Treg cells, thereby promoting the costimulation and amplification of tumor-specific CTLs [479]. To further improve the efficacy of checkpoint inhibitor antibodies, bifunctional antibody ligands have been produced that consist of antibodies targeting CTLA-4 or PD-L1 fused into the TGFβ receptor II extracellular domain (TGFβRIIecd), where TGFβRIIecd isolates TGF-β secretion in the TME, while checkpoint inhibitor antibodies consume Tregs and promote CTL costimulation [480]. This dual strategy may be more effective against cancers resistant to checkpoint inhibitors alone. As an alternative to TGF-β isolation, tumor-reactive T cells can be transduced with dominant negative TGF-β receptor-II (DNTGf-βrii) to produce TGF-β-resistant antitumor T cells. TCR-T cells expressing dnTGFβRII showed complete tumor regression and prolonged survival in mouse models of advanced and invasive prostate cancer [481, 482]. To take advantage of the high concentration of TGF-β in the TME, a study fused the extracellular domain of TGF-β receptor II (TGF-β RII) into the intracellular domain of 4-1BB. This strategy converted the immunosuppressive effect of TGF-β into an immunostimulatory signal. Coexpression of this transgene receptor produces additive effects and improves the amplification, persistence, tumor lysis, and selective antitumor activity in vivo [483]. Similar to TGF-β, Fas ligand-mediated T-cell death signals that are highly expressed in the TME can also be transformed into pro-survival signals via CSR. These signals enhance the proliferation and antitumor function of engineered T cells by fusing the Fas extracellular domain with the 4-1BB intracellular domain [484].

### (三)、Insufficient production of neoantigen-specific T cells

Immunotherapies, including vaccination, adoptive TIL and TCR-T cells, and ICBs, all rely on neoantigen-specific T cells [163, 459]. Direct detection of neoantigens presented by tumor cells may be the most effective way to build neoantigen-reactive T cells, ensuring that they recognize new epitopes in the body [175]. However, the implementation of TIL therapy will be hampered by the scarcity of fresh tumor samples and low TIL cold tumors. First, the acquisition of TILs requires invasive surgery to remove resectable lesions, making it suitable for only some patients. Second, inhibition of the TME in cold tumors may reduce the efficiency and number of neoantigen-specific T cells derived from TILs. Readily available peripheral blood may be a suitable source for generating large numbers of neoantigen reactive T cells for ACTs. However, it should be emphasized that large amounts of in vitro amplification can increase both the further differentiation of T cells and the false-positive neoantigen response of T cells. Most cancer patients cannot be treated with TILs due to inadequate TCR libraries [485–487]. Efforts are underway to develop effective strategies to isolate and rapidly amplify neoantigen-specific T cells, which may benefit neoantigen-based behavior. TILs contain a large number of neoantigen-reactive T cells, making them a valuable source of T lymphocytes for ACT [237, 255, 391]. Currently, mature DC or EBV-transformed B-cell lines that transfect APCs with peptide pulses or TMGs are used to present antigens to T cells [488, 489]. mRNA transfection of DCs expressing neoantigens was also used to activate autologous naive CD8^+^ T lymphocytes from healthy donors who had not been exposed to the immunosuppressive environment of the tumor host. However, TCRs triggered by APCs that encode mRNA or synthesize peptide pulses may not endogenously recognize antigen-presenting tumor cells [490]. Naive and very early memory T cells with stem-like properties can be generated using induced pluripotent stem cell (iPSC) techniques. Immature T-cell lines derived from iPSCs can now be further differentiated into neoantigen-specific T cells based on a unique three-dimensional thymus organ culture system that fully generalizes disease in vitro [491, 492]. Another benefit is that iPSCs can be cloned from a single neoantigen-specific αβ and re-differentiated into a large number of CTLS. In the near future, iPSCs from single CTL clones will be used to generate sufficient numbers of neoantigen-specific TCR-T cells. These cells remain naive and contain an endogenous state of TCR, thereby significantly increasing the efficacy of neoantigen-based immunotherapy [493].

### (四)、The singleness of therapeutic targets

In current clinical studies on TCR-T cells, only TCR-T cells attacking a single epitope have been utilized [176]. Heterogeneity is an important characteristic of tumors. This means that this unique target of T-cell attack may be decreased or even disappear, leading to T-cell therapy failure [260, 323]. The use of single TCR-T cells may also be an important reason for the poor results of such clinical trials. To achieve better tumor treatment results, combination therapy with TCR-T cells targeting different sites must be used to prevent immune escape [10].

### (五)、Immunotherapy resistance

The mechanism of primary and secondary resistance to TCR-based immunotherapy may manifest as the low expression or heterogeneity of target antigens in tumor cells or the intrinsic resistance of tumor cells to T-cell-mediated cytotoxicity [5, 397]. The main mechanism of resistance to TCR-T-cell therapy is the loss or reduction of MHC-I/II class molecules on the surface of tumor cells, which prevents TCR-T cells from recognizing target epitopes [85, 126, 141, 161, 353, 395].

### (六)、The targeted antigen is mainly HLA-A2-restricted

Most of the antigen recognition in clinical trials of TCR-T cells is HLA-A2-mediated [119, 494, 495]. Although HLA-A2 is the main MHC molecule in the population, the positive rate is high (approximately 1/3 of the Chinese population expresses HLA-A2). However, TCR-T cells that rely solely on the recognition of HLA-A2 presenting antigens are not applicable in a significant number of patients. The search for only HLA-A2-dependent TCRs also limits the search for targeted antigens [496, 497]. Many of the antigens suitable for TCR-T-cell therapy may be presented by other MHC molecules [263, 315]. Monoclonal or oligoclonal neoantigen-reactive T cells made by amplification would also be an alternative route [10]. Therefore, other HLA-polypeptide tetramers must be developed to broaden the search for antigenic and reactive TCRs.

### (七)、The safety of TCR-T-cell therapy

Safety is an issue that must be addressed before TCR-T cells can be used in clinical therapy [498, 499]. There have been cases of death in clinical trials of TCR-T cells. The reason may be that the enhanced affinity of TCR leads T cells to recognize autoantigens with high homology and then attack normal cells and tissues, eventually leading to death [500, 501]. Thus, the balance between functionality and security must be considered in TCR transformation. The safety of TCR-T-cell therapy should be carefully determined by sequence alignment, cell experiments and animal experiments [502]. Another way to ensure the safety of TCR-T cells is to introduce the suicide gene. TCR-T cells with the suicide gene can perform the killing function normally. However, after the injection of suicide gene activating drugs, T cells undergo apoptosis to avoid the nonspecific killing of T cells [503–505].

### 九、Advances in TCR-T-cell therapy

In clinical studies, the safety and efficacy of TCR-T-cell therapy have shown major problems. To solve these problems, in the field of TCR-T-cell research, the search for safe and effective target antigens to improve the affinity and efficiency of TCRs has been the focus. However, it is worth noting that in recent years, an increasing number of studies have been conducted to search for reactive TCRs using neoantigens as targets, and good results have been achieved.

### (一)、Identification of neoantigen reactive TCRs

Neoantigens are abnormal peptides produced by genetic mutations. These antigens are often tumor specific, making them good therapeutic targets [163]. In recent years, with the advancement of sequencing technology, it has become possible to conduct high-throughput sequencing of tumor tissue and normal tissue to look for neoantigens [426, 506, 507]. After the neoantigen mutation site is identified, the neoantigen reactive TCR can be found by coincubation with tumor-infiltrating T cells by using the presenting cells to express multiple antigenic peptides at different locations of the mutant epitopes [159, 350, 508]. In addition, the expression of coinhibitory molecules such as PD-1, 4-1BB and ICOS is also helpful in searching for neoantigen-reactive TCRs [244, 280, 375]. Targeting neoantigens is an attractive therapeutic strategy, but it falls into the category of individualized therapy and is costly. At present, it is not the mainstream strategy of TCR-T-cell therapy [176]. However, TCR-T-cell therapy targeting neoantigens will be a direction for development in the future, and it could even be the main direction of research [260].

### (二)、The therapeutic effect of CD4^+^ TCR-T cells

CD8^+^ T cells are traditionally considered to be the primary tumor killer cells [188]. Therefore, the role of CD4^+^ T cells in tumor therapy has not received much attention. In recent years, however, it has been found that CD4^+^ T cells also play an important role in tumor killing [280, 282, 348]. In the identification of TCRs, we have always been searching for MHC-I molecular-restricted TCR sequences. The transfer of such TCRS into CD4^+^ T cells can also promote the function of the latter. The role of CD4^+^ T cells in tumor control has also been directly demonstrated in clinical trials. A reduction in tumor charge was also observed after patients received neoantigen-reactive CD4^+^ T cells. However, due to the lack of the stabilizing effect of the CD4 molecule, it is difficult for these CD4^+^ T cells to play a long-lasting role in the body [263, 509]. In general, it is thought that the recognition of antigens by TCR is MHC-restricted, that is, it recognizes antigens presented by either MHC-I or MHC-II molecules [126]. However, studies have shown that some TCRs can recognize both MHC-I and MHC-II molecular presented antigens. This means that such TCRS may be stabilized by both CD8 and CD4. The therapeutic effect of CD4^+^ TCR-T cells will be improved by using such TCRs for treatment [510].

### (三)、The selection of TCRα and β chain connection sequences

The TCR consists of alpha and beta chains. In natural T cells, alpha and beta chains are encoded by two loci, each transcribed and translated, and then the TCR is assembled [511]. However, in TCR-T cells, to synthesize functional exogenous TCRs, it is necessary to consider introducing both α-chain and β-chain genes into the same T cell. In this process, two vectors or two promoters in the same vector can be considered to express the α or β chains. However, this may lead to an imbalance in the α chain and β chain expression of exogenous TCR, increasing the probability of TCR mismatch [232, 512]. Therefore, a better solution is to make the α and β chains under the control of a promoter by using the linking sequence to ensure the balance of molecular translation. The internal ribosome entry site sequence (IRES) is a widely used bicistronic junction sequence that enables coexpression of the IRES. However, the efficiency of IRES-mediated translation is low, resulting in low expression levels of genes located behind IRESs. If IRES sequences are used to connect the alpha and beta chains of TCRs, mismatches may still occur [513–515]. In the TCR expression system, the 2A peptide sequence derived from picornavirus or porcine enterovirus is a good choice. Peptide 2A has a self-cutting function. In other words, when the α-β chain is connected by the 2A sequence, the two chains are transcribed and then broken into separate peptide segments after translation. The amount (mole) of TCR chain can be expressed by using the 2A sequence to reduce the probability of mismatch. Notably, the function of TCR is not affected by the 2A binding sequence despite the addition of several additional amino acids to the α and β chains of TCR [515, 516].

### (四)、Improvement of the surface expression efficiency of the TCR membrane

When a sufficient number of TCRs recognize and bind to pMHC, T cells are activated to function. The amount of TCR required for T-cell activation can be reduced by costimulation. However, the continued activation of T cells still depends on the number and affinity of TCRs on the cell surface [517, 518]. The assembly of TCR and the localization of the membrane surface are complicated processes. The translated α and β chains are assembled to form heterodimers, which bind to multiple CD3 subtype molecules (γ, δ, ε, and ζ). The amount of CD3 molecules, especially of the ζ subtype, is constant in the cell. If a complete complex cannot be formed with the CD3 molecule, the excess TCR is degraded [511, 519]. The problem is particularly acute in TCR-T cells. The use of a strong promoter and 2A sequence can ensure the efficient expression of exogenous TCR in host cells. However, the number of TCRs that can bind to CD3 is limited, and there is competition to bind to endogenous TCRs. This will result in the degradation of a large number of TCR transgene products that are unable to form functional TCR-CD3 complexes [516, 520, 521]. To solve this problem, TCR and CD3 molecules can be expressed together. Animal experiments have shown that when TCR is coexpressed with CD3 subtype molecules, the expression of TCR on the T-cell surface increases by tens of times. This increases the affinity of T cells to target antigens while improving the efficiency of tumor clearance and memory response [521]. In addition, appropriate modifications of TCR coding sequences, such as the deletion of unstable mRNA sequences and splicing sites, can also upregulate the expression of TCR transgenes and enhance the anticancer activity of TCR-T cells [513, 522].

### (五)、Selection of the TCR constant region (C region)

The C region of TCRs plays an important role in the correct pairing of alpha and beta chains. If the C region of exogenous TCR can be made different from the C region of the host itself, the correct heterodimer formation of exogenous TCR can be guaranteed [520]. The experimental observation showed that the expression efficiency of mouse TCR in human T cells was higher than that of human TCR, suggesting that mouse TCR could be correctly assembled and bind to CD3 molecules [523]. The C region of mouse TCR plays an important role in this phenomenon. Based on this orientation, a hybrid TCR containing the human V region and mouse C region was developed and studied. The results showed that the function of the hybrid TCR was stronger than that of the fully human TCR in human T cells. These results indicated that the C region of mice ensured the preferential pairing of exogenous mixed TCR itself and the stability of the mixed Tcr-CD3 complex while improving the membrane surface expression efficiency of TCR [524–526]. Although the introduction of mouse region C induces immune rejection, the immunogenicity of the mixed TCR can be significantly reduced by modifying a few amino acids in mouse region C. In addition to introducing mouse sequences, amino acid sequences in the C region can also be modified to improve the accuracy of TCR pairing. Cysteine plays an important role in the pairing and stabilization of the alpha and beta chains of TCR [527]. Using a point mutation technique, the threonine at position 48 of the alpha chain and the serine at position 57 of the β chain were replaced with cysteine, forming an additional disulfide bond in the C region. This change enhanced the pairing efficiency and stability of the exogenous α and β chains, reduced the occurrence of mismatch, increased the expression of exogenous TCR on the cell surface, and enhanced the antigen-specific response. Studies have shown that this strategy can reduce the autoimmune pathological reactions caused by TCR mismatch [528].

### (六)、Other strategies to reduce TCR mismatch

Endogenous TCR expression can be reduced using siRNA or CRISPR/cas9 techniques. This can not only prevent TCR mismatch but also reduce the competition between TCR and CD3 molecules [355, 516]. Several studies have shown the feasibility of this strategy. For example, T cells have coexpressed MAGE-A4-specific TCRs (whose codon-optimized C region is different from that of the wild type) and siRNA targeting conserved sequences in the C region of wild-type TCRs. The results showed that the surface expression of the TCR transgene membrane in human T lymphocytes transduced by the MAGE-A4-TCR/siRNA vector was upregulated [529, 530]. In addition, the transfer of the TCR gene into γδT cells can also prevent the mismatch of endogenous TCR. However, γδT cells did not express CD4 and CD8 molecules. The use of γδT cells to express functional α/βTCR requires simultaneous transduction of CD4 or CD8 molecules to enhance the antigen-specific immune response of γδT cells [531–533].

### (七)、Enhanced T-cell activation signals

CD3ζ and costimulatory or coinhibitory molecules are the main signaling molecules that directly affect the activity of TCR-T cells [534]. The function of TCR-T cells is affected by altering the expression and function of CD3ζ and/or costimulatory/coinhibitory molecules. By coexpressing CD3ζ in series with the α and β chains of TCR, it can increase the membrane surface expression of TCR and enhance the function of antigen-specific T cells [535]. In addition, the costimulatory signal CD28 can be introduced into the TCR sequence to enhance TCR signal transduction. This method requires the complete removal of the C region and its replacement with the transmembrane region of CD28. Therefore, such TCRs do not pair with endogenous TCRs and have a stronger activation capacity. However, this method directly connects Vα and Vβ through flexible sequences, which destroys the original TCR sequences and may not be suitable for all TCR sequences, limiting the application of this modification strategy [373, 536]. In recent years, PD-1 has received great attention. If the PD-1 inhibition of T cells can be removed, the function of TCR-T cells can be effectively improved. Currently, CRISPR/Cas9 can be used to knockdown PD-1 expression or express specific antibodies to block PD-1. It can not only make TCR-T cells have tumor killing specificity but also block the cosuppression signal to avoid the suppression of tumor killing function [537–541].

### (八)、Improved TCR affinity

The affinity between TCR and antigen determines the ability of T cells to recognize and kill tumor cells, which is the main focus of TCR-T cell research [350]. (1) The isolation of high-affinity TCR from artificial nontolerant environments is performed as follows: This approach utilizes humanized mice to produce high-affinity T cells [542]. CD8^+^ T cells in the body that have a high affinity for the tumor-associated antigen p53 are often deleted. Immunizing transgenic mice expressing human HLA-A2 with human p53 peptide induced amplification of CD8^+^ T cells with high affinity for human p53 [543, 544]. When isolated from these high-affinity TCR sequences, these peptides can be used to redirect human CD8^+^ or CD4^+^ T cells. Using the same method, T-cell clones with high affinity for other tumor-associated antigens, including MDM2, CEA, and gp100, have been isolated [545–547]. (2) The isolation of high affinity MHC-restricted TCR from MHC-mismatched donors can be performed as follows: T-cell clones with high affinity for the HLA-A2 presenting antigen can be isolated from the natural lymphocyte pool of HLA-A2 negative donors. Using this method, human T-cell clones with high affinity for tumor-associated antigens, such as cyclin D1, WT1, and MDM2, were isolated, and these high-affinity TCR genes were cloned into vectors for gene therapy [548–551]. In mouse models, human T cells with allogeneic HLA-A2 antigen-specific TCR gene transduction were shown to clear tumor cells [549, 552]. (3) TCR sequences can be modified to improve affinity as follows: Improving affinity by changing the sequence of the TCR is an effective way to improve the effects of TCR-T-cell therapy. The CDR region responsible for binding to pMHC determines the affinity of TCR, and modification of the CDR region is expected to improve T-cell function [520, 527, 553]. Substituting an amino acid at site 107 in TCR has been shown to improve the stability of the CDR3β ring structure and increase the antigenic specificity of TCR [554, 555]. In screening TCRs with high affinity mutations, point mutation techniques can be used to construct a variety of TCR α chain and β chain libraries. The assembled TCRSs can then be displayed using phages, yeast, or T cells, and affinity screening is performed [556–558]. MHC- polypeptide tetramers are an important tool in affinity screening. High affinity TCRs can even be screened from natural TCRs by using tetramers. The high-affinity TCRs screened can theoretically enhance the antigenic reactivity of T cells [170, 559]. However, some studies have shown that T cells transduced with high-affinity TCRs do not respond to antigens or even have negative reactions. Another concern is that high-affinity TCRs can misrecognize antigens, resulting in off-target effects [350, 560, 561].

### (九)、CD4^+^ T cells are dependent on MHC-I molecule-restricted TCRs

Early studies on TCR-T cells focused on how to construct antigen-specific CD8^+^ T cells [176]. Currently, the influence and role of CD4^+^ T cells is also being considered. CD4^+^ T cells recognize the MHC-II class molecular delivery of antigenic peptides. Unlike CD8^+^ T cells, which mainly exert cytotoxic effects, CD4^+^ T cells mainly regulate the adaptive immune system, enhance the function of CD8^+^ T cells, and induce the long-term memory of T cells [267, 340, 341]. Although the isolated TCRs with high affinity for tumor antigens were mostly MHC-I molecule restricted, they performed best in the presence of CD8 coreceptors [282]. Studies have shown that these TCRs can function in CD4^+^ T cells even in the absence of CD8 coreceptors. Tumor-specific CD4^+^ T cells can be generated by using MHC-I molecular restrictive TCR, which enhances the tumor-killing ability of specific CD8^+^ T cells. The reason for this phenomenon may be related to the secretion of various immune factors by CD4^+^ T cells [344, 349, 562]. In addition, antigen-specific CD4^+^ T cells have also been used to treat tumor patients [563–565]. These results suggest that attention should be given to the role of CD4^+^ T cells in TCR-T-cell therapy.

### (十)、Prolonging the survival time of TCR-T cells in vivo

A common challenge faced by adoptive cell therapy is the survival time of the transfused cells in vivo [256]. Studies of TCR-T cells and CAR T cells have demonstrated that T-cell infusion can lead to the formation of memory and these cells can survive long-term in vivo [176, 257, 398]. However, the number of such cells is too small to play a role in the treatment of solid tumors. At present, the common ways to maintain the survival of T cells include the administration of exogenous IL-2 and the removal of lymphocytes from the body by radiotherapy or chemotherapy [255–257]. Among them, IL-2 injection has relatively obvious toxicity, especially in the application of high doses, and adverse reactions are more obvious. The adverse effects of IL-2 limit its use. To address this limitation, researchers have tried to express IL-2 in T cells, but the results were not good [566]. Another approach is to use chemoradiotherapy to eliminate lymphocytes, reduce the number of endogenous T cells, and avoid endogenous T cells competing for cell growth factors [567]. Animal experiments have shown that without preconditioning, transfused T cells cannot survive or clear tumors. Lymphocyte elimination preconditioning has become the standard practice for infusion cell therapy, including for TCR-T cells [268]. The TCR sequence can also be transferred into less differentiated T cells or memory T cells. In addition to treating T cells directly, TCRs can also be transferred into hematopoietic stem cells (HSCs). Transgenic HSCs can develop into mature CD8^+^ T cells through positive selection and produce a rapid antigen-specific response [568, 569]. In addition, it is possible to prolong the survival of TCR-T cells in vivo by influencing T-cell differentiation through metabolically regulated drugs, such as metformin [570].

### 十、Summary and outlook

Neoantigens play a key role in cancer immunotherapy, including cancer vaccines, ACTs, antibody-based therapies, and ICBs [571]. Therapeutic strategies targeting these cancer-specific neoantigens without destroying normal tissue provide a strong theoretical basis to support the relevance of neoantigens in supporting clinically successful immunotherapies. Many initiatives are underway to develop personalized or off-the-shelf anticancer drugs based on neoantigens. However, to address the timing and economics of advanced personalized neoantigen immunotherapy, experimental and theoretical improvements are needed, including effective patient recruitment, optimized sequencing techniques and neoantigen prediction algorithms, and off-the-shelf therapies targeting common neoantigens [272].

Effective patient recruitment is key to neoantigen-based immunotherapy. First, early excision may give clinicians more time to carefully design, produce and test neoantigen-based therapeutics to improve clinical outcomes. Second, early excisions make it easier to select patients who may be eligible for off-the-shelf therapies, including vaccines, TCR or TCRm antibodies that target a well-defined cancer-driving mutation. Third, cancer treatments, including chemotherapy, radiation, and ICBs, can stimulate the over-differentiation of T cells. Immediately isolating autologous T cells, or TILs, as soon as a patient is diagnosed with cancer allows for the highest quality and least differentiated T cells to be collected from the patient. In addition, neoantigen-based therapy in patients who were recruited early can allow for the effective infusion of high-quality neoantigen-based cell products and reduce the severity of comorbidities caused by advanced metastatic cancer clones.

Accurate identification of immunogenic neoantigens and their homologous TCRs is a key and rate-limiting step in the development of personalized cancer immunotherapy [163]. Immunogenic neoantigens can be identified by the immunogenomics methods of constructing virtual peptides based on NGS data and the immunolabeling methods of analyzing MHC-loaded peptides using MS. Genome and transcriptional sequencing data are also combined with MS maps of HLA-related peptides to improve the sensitivity and specificity of neoantigen identification [292]. However, neoantigen-based therapies may be quite affordable due to the application of more economical high-throughput sequencing and powerful deep learning algorithms. Comprehensive and efficient one-stop computational workflows or classification benchmarks available in computerized neoantigen assay methods are still necessary for clinical applications. Efficient one-stop computing methods can also use large data queues to determine the potential of neoantigens as biomarkers for patient prognosis or ICB response prediction. Most critically, the accuracy of these epitope prediction methods should also be confirmed by thorough immune surveillance in early clinical studies to facilitate the development of neoantigen-based cancer therapies [163, 171, 253]. In addition to these computerized methods for predicting immunogenic neoantigens and homologous TCRs based on high-throughput sequencing data, several T-cell antigen discovery strategies have recently been developed to unbiasedly identify immunogenic neoantigens. A variety of pMHC libraries have been established, including yeast display libraries, SABRs, BATLLES, etc., which allow the flexible and extensible screening of antigen epitopes. By relying on the physiological activity of T-cell killing rather than evaluating the binding affinity of TCR-pMHC, T-Scan is able to query a significantly larger antigen space independent of predictive algorithms [171, 229, 230, 572]. Therefore, the simplicity and scalability of T-cell ligand discovery techniques will facilitate study of the immunogenicity of candidate neoantigens and contribute to the development of new neoantigen-based immunotherapies.

Off-the-shelf precision immunotherapies targeting common neoantigens is another possible strategy to overcome the time and funding issues in individualized therapy based on personalized neoantigens. Common neoantigens shared between patients will be produced by driver genes or TSGs with hot mutation polypeptides presented by relatively common HLA alleles. Common neoantigens are more likely to be clonally conserved in metastatic tumors and to reappear systematically in patients. Many general-purpose therapeutic techniques readily target common neoantigens, including vaccines, BSABs, CTLS, and adoptive metastasis of TCR-T cells. Subsequently, TCR libraries that specifically target shared neoantigens in an HLA-specific manner are developed for advanced cancer patients. As more common neoantigens and corresponding TCRs are discovered, more patients with frequent genetic changes that lead to cancer will benefit from the public neoantigen response TCR library. In addition, the widespread use of cancer genome sequencing and neoantigen prediction methods will help to match patients to treatments that target common neoantigens in their tumors [5, 573–575]. Therefore, this off-the-shelf strategy based on common neoantigens is expected to shorten the time required for neoantigen identification and widespread T-cell culture, increasing the use of neoantigen-based therapies in a significant proportion of patients.

In addition to neoantigens produced by spontaneous mutations during carcinogenesis, some covalent molecules can induce the production of tumor-specific public neoantigens by modifying hotspot residues in tumors with highly repetitive somatic mutations. Covalent KRAS-G12C inhibitors, such as ARS1620, irreversibly modify mutant cysteines. Hapten peptides carrying covalently linked small molecules can be presented on the cell surface via MHC-I. Hapten peptide-MHC complexes can act as tumor-specific neoantigens to trigger cytotoxic T-cell responses [89, 576, 577]. Based on this principle, mutant tumor suppressor proteins can be targeted by a new class of molecules. These molecules induce neoantigen production and trigger specific immune responses by covalently modifying hotspot residues, such as TP53 Y220C and TP53 R273C [578, 579]. Thus, the range of tumor-specific neoantigens suitable for therapeutic targeting can be significantly expanded by specifically modifying haptens of mutated cancer proteins rather than acting as drug inhibitors.

These neoantigens provide powerful targets for tumor vaccines that not only accurately eliminate residual tumor lesions but also effectively target distant metastatic cells due to their systemic properties. Personalized neoantigen vaccines are produced according to the individual tumor through the following steps: the collection of tumor tissue and normal samples, sequencing and analysis of unique mutations, prediction and validation of immunogenic neoantigens, and vaccine design and production. A variety of platforms, including peptides, nucleic acids and DCS, can be used to develop vaccines based on predicted personalized or matched shared neoantigens. Neoantigen vaccines based on peptides, RNA and DNA are feasible, safe and economical vaccines [192, 292, 382, 386]. However, most patients cannot reliably produce a substantial neoantigen-specific CD8^+^ T-cell response by peptide-based neoantigen vaccines. All active components of tumor vaccines, such as neoantigens, formulations, and delivery systems, are constantly being improved. Synthetic self-amplified mRNAs (samRNAs) containing replicase genes encoding RNA-dependent RNA polymerase (RdRp) have attracted much attention due to their higher and longer-lasting antigenic expression than traditional mRNAs. In vivo expression of vaccine neoantigens can also be enhanced by vectors that naturally carry genetic instructions, including adenoviruses (Ads), retroviruses, and adeno-associated viruses (AAV). In addition, various nanoparticles are formed, such as lipid nanoparticles, exosomes, viral-like particles, cage protein nanoparticles, bacterial membrane material-based nanocarriers, high-density lipoprotein mimic nanodisks, polymers and polymer nanoparticles, to enhance transport and tissue penetration capabilities and improve the immunogenicity of personalized vaccines. Compared with viral vectors, nanoparticles can also effectively deliver vaccines and immune adjuvants together to lymphatic organs to increase neoantigen presentation [292, 382, 580].

Monocytes or hematopoietic progenitor cells isolated from blood loaded with tumor neoantigens in vitro can effectively improve the anticancer effect of neoantigen vaccines. Autologous DCs can be loaded with neoantigens in the form of peptides, RNA and DNA [382, 581]. Compared with the time-consuming and costly sequencing and computational analysis of patient-specific neoantigens, autologous dendritic cell (DC) vaccines with whole-tumor lysate (WTL) induce a T-cell-mediated antitumor response in vitro and are a more convenient and economical method to induce neoantigen-specific immune responses. The entire tumor cell contains both MANAs and nonmutated TAAs, which may overcome potential immune escape and resistance mechanisms. However, the higher abundance of nonimmunogenic autoantigens may limit the ability of neoantigens to induce immune responses. Various immunosuppressive factors also exist in WTL, which inhibit DC maturation and T-cell activation [582–585]. To overcome these problems, extracellular vesicles (EVs) produced by tumor cells have recently been shown to be a vaccination platform that supports DC maturation and neoantigen presentation. Tumor cell-derived EVs can deliver tumor antigen libraries to DCs and promote the cross-presentation of neoantigens. EVs also have high levels of immune stimulators, which can trigger DCs to release natural immune signals. In addition to tumor cell-derived EVs, DC-derived EVs can also serve as neoantigen-presenting units for immune cells. EV-based vaccines can transform "cold" tumors into "hot" tumors by modulating the tumor immune microenvironment and systemic immune response [586–589]. Thus, EVs may provide an alternative form of neoantigen-based cancer vaccine that might be administered orally.

Although neoantigen-based immunotherapy has shown promising results in early preclinical and clinical studies, significant progress remains to be made, especially in patients with epithelial malignancies. Cancer cells have evolved built-in defenses to evade immune recognition at every stage of the cancer immune cycle. Given the complex mechanisms of immune escape in cancer, combination therapies that simultaneously target different stages of the cancer immune cycle may be more effective. Cell death following chemotherapy, radiotherapy, targeted therapy, photodynamic therapy, and oncolytic virus therapy will promote the production and release of neoantigens, thereby further enhancing the antitumor immune cycle [10, 590]. The use of IFN-α, GM-CSF, anti-CD40, TLR agonists, and STING agonists promoted neoantigen presentation [591–594]. To promote the infiltration of immune cells into the tumor, TME modifications and intracellular cytokines can be used. ICBs and IDO inhibitors will also alter immunosuppressive TMEs to enhance neoantigen-based immunotherapy [322, 378]. Nano and EV drug delivery systems have recently been used as an integrated platform for the simultaneous administration of many drugs or therapeutic drugs that work synergistically to activate different stages of the cancer-immune cycle, reverse immunosuppression and create immune support TMEs [595, 596]. These combination strategies using therapeutic agents with different mechanisms of action induce a strong, effective, durable, and tumor-specific immune response in cancer patients.

## 十一、Conclusion

The viability of TCR-T cells in treating tumors has been demonstrated. At present, although there are still some problems in TCR-T-cell therapy, more attention should be given to the powerful clinical application prospects of this kind of therapy. With advances in tumor immunology and genetic engineering, TCR-T-cell therapy will become more individualized. At present, TCR-T cells are mainly focused on "universal targets" for treatment. When used for treatment, TCR-T cells only consider the expression of antigen and MHC molecules. When these two conditions are met, they are used for the treatment of multiple patients. However, the actual situation of these patients varies greatly, and the low selectivity of TCR-T-cell therapy may be related to poor or ineffective treatment response. Studies have shown that the development of TCR-T cancer immunotherapy that targets certain tumors with intracellular antigens has become a research hotspot in recent years. If individual TCR-T cells can be designed according to the expression of patients' tumor-associated antigens or even neoantigens, it is possible to significantly improve the efficacy and safety of treatment. The maturation of sequencing technology and advances in cell culture technology have laid the foundation for this scenario. On the other hand, the use of under-differentiated T cells or hematopoietic stem cells to produce TCR-T cells with longer in vivo survival will also improve the effectiveness of such treatments. In addition, TCR-T cells can be used in combination with immune checkpoint blockers to improve the efficacy of TCR-T cell treatments. In summary, TCR-T cells will play an increasingly important role in tumor therapy, and their development will bring more hope to tumor patients.

## Data Availability

All the data that support the findings of this study are available from the corresponding authors upon reasonable request.
